# The short-chain fatty acid receptors Gpr41/43 regulate bone mass by promoting adipogenic differentiation of mesenchymal stem cells

**DOI:** 10.3389/fendo.2024.1392418

**Published:** 2024-09-19

**Authors:** Friederike Behler-Janbeck, Anke Baranowsky, Timur A. Yorgan, Michelle Y. Jaeckstein, Anna Worthmann, Marceline M. Fuh, Karthikeyan Gunasekaran, Gisa Tiegs, Michael Amling, Thorsten Schinke, Joerg Heeren

**Affiliations:** ^1^ Department of Biochemistry and Molecular Cell Biology, University Medical Center Hamburg-Eppendorf, Hamburg, Germany; ^2^ Department of Trauma and Orthopaedic Surgery, University Medical Center Hamburg-Eppendorf, Hamburg, Germany; ^3^ Department of Osteology and Biomechanics, University Medical Center Hamburg-Eppendorf, Hamburg, Germany; ^4^ Institute of Experimental Immunology and Hepatology, University Medical Center Hamburg-Eppendorf, Hamburg, Germany

**Keywords:** G protein-coupled receptors, Gpr41/43, short-chain fatty acids (SCFAs), acetate, osteoblasts, bone formation, adipogenesis

## Abstract

Bone is a dynamic tissue that is constantly remodeled throughout adult life. Recently, it has been shown that bone turnover decreases shortly after food consumption. This process has been linked to the fermentation of non-digestible food ingredients such as inulin by gut microbes, which results in the production of the short-chain fatty acids (SCFAs) acetate, propionate and butyrate. SCFAs exert various metabolic functions, which in part can be explained by activation of G protein-coupled receptors (Gpr) 41 and 43. However, the potential relevance of a SCFA-Gpr41/43 signaling axis for bone metabolism has not been established. The aim of our study is to investigate the role of Gpr41/43 in bone metabolism and osteogenic differentiation of mesenchymal stem cells. For this purpose, we analyzed the skeletal phenotype of wild type controls (WT) and Gpr41/43 double knockout (Gpr41/43 dKO) mice fed either a chow or an inulin-enriched diet. In addition, we isolated bone marrow derived mesenchymal stem cells from WT and Gpr41/43 dKO mice and differentiated them into osteoblasts in the absence or presence of acetate. MicroCT scanning of femoral bones of Gpr41/43 dKO mice revealed a significant increase of trabecular bone volume and trabecular compared to WT controls. Treatment of WT bone marrow-derived osteoblasts with acetate resulted in decreased mineralization and substantial downregulation of bone formation markers such as *Phex*, *Ptgs2* and *Col1a1*. Notably, this effect was strongly attenuated in differentiated osteoblasts lacking Gpr41/43. Inversely, acetate supplementation resulted in higher levels of adipocyte marker genes including *Pparg*, *Lpl* and *Adipoq* in bone marrow-derived cells from WT mice, an effect blunted in differentiated cells isolated from Gpr41/43 dKO mice. Overall, these data indicate that acetate regulates bone architecture via SCFA-Gpr41/43 signaling by modulating the osteogenic versus adipogenic differentiation of mesenchymal stem cells.

## 1 Introduction

Bone is constantly remodeled throughout an organism’s life and this physiological process mainly involves the antagonistic roles of bone-forming osteoblasts and bone-resorbing osteoclasts (1, 2). Both cell types differ fundamentally in terms of progenitor cells, activity and regulatory molecules. More specifically, osteoblasts originate from mesenchymal progenitors and act in large groups of cells secreting an extracellular matrix (osteoid) that gradually mineralizes (1). A subset of osteoblasts undergoes terminal differentiation into osteocytes forming a cellular network within the mineralized bone matrix, which modulates bone remodeling and mineral homeostasis (3). In contrast, osteoclasts are derived from the fusion of hematopoietic cells that belong to the monocyte/macrophage lineage and resorb bone matrix by extracellular acidification and secretion of matrix-degrading enzymes (4). A dysbalance in bone remodeling towards bone resorption causes bone loss that can result in osteoporosis, a detrimental disease characterized by low bone mass and microarchitectural deterioration (5). Osteoporosis can be treated by two entirely different pharmacological approaches: The anti-resorptive therapy leads to an inhibition of either osteoclast differentiation (Denosumab) or osteoclast function (bisphosphonates) (6, 7). Osteoanabolic treatment regimens, such as the daily injection of parathyroid hormone fragment (Teriparatide) or the monthly injection of a more recently developed antibody against Sclerostin (Romosozumab), promote bone formation. However, the repetitive injection of osteoblast-activating drugs requires patient’s compliance and is associated with high costs and adverse effects (8, 9). Accordingly there is an urgent need to establish safe and cost-effective osteoanabolic therapies.

The process of bone remodeling and maintenance of the skeleton is an energy-demanding process and requires metabolic adaptations by osteoblasts (10). One major component of bone remodeling is the intake of nutrients. Interestingly, it has been reported that bone turnover decreases shortly after food consumption (11, 12). Malnutrition, such as that caused by anorexia nervosa, is also associated with reduced bone mineral density and an increased risk of fractures (13, 14). On the other hand, high intake of plant fibers seems to be beneficial for bone health (15, 16). Dietary effects are at least partly determined by gut microbiome composition. The absence of gut microbiota in animal models influences bone remodeling, trabecular bone mineral density and bone volume fraction (17–19). It was also shown that gut microbiota modulate bone growth in the neonatal phase (20). Furthermore, an imbalance in gut microbiota has been observed in patients suffering from osteoporosis (21).

In recent years it has become apparent that not compositional changes *per se* but rather the generation of metabolites from gut microbes have an impact on host metabolism. For instance, bacterial enzymes modify host metabolites such as bile acids, which can re-enter the systemic circulation via the enterohepatic circulation (22). In the context of bone metabolism, we recently described that increased concentrations of specific bile acids are associated with osteoporosis in patients with primary sclerosing cholangitis, linking alterations in bile acid metabolism to a bone phenotype (23). Furthermore, intake of fiber-rich, non-digestible carbohydrates results in anaerobic fermentation and the production of SCFAs (24, 25), which can activate immune cells but also distant parenchymal cells by ligation of G protein-coupled receptors (26). In this context, we recently found that energy-dense nutrients result in mucosal and systemic immune depression, an effect that can be rescued by supplementation with SCFAs (27, 28).

SCFAs serve as energy sources and affect their target cells either by free diffusion, carrier-mediation or receptor transfer (29–31). Growing evidence supports the idea that SCFAs are an important metabolite class and exert essential physiologic effects on many organs including adipose tissue, liver, brain, but also immune cells and contribute to health (32–34). The beneficial effect of SCFAs on bone remodeling has been linked to increased calcium and magnesium absorption after SCFAs supplementation (35, 36). Additionally, incubation with supraphysiological butyrate concentrations can directly modulate the function of osteoclasts and osteoblasts (37). Next to their roles as energy sources or posttranslational modifications, SCFAs activate the G protein-coupled receptors Gpr41 and Gpr43. However, the relevance of this signaling axis for bone metabolism is unclear. In the present study, we investigated the role of these SCFA receptors in bone metabolism and analyzed the impact of SCFAs on activation of Gpr41 and Gpr43.

## 2 Materials and methods

### 2.1 Animal experiments

All mouse experiments were approved by the Animal Welfare Officers of University Medical Center Hamburg-Eppendorf (UKE) and Behörde für Justiz und Verbraucherschutz (formerly Behörde für Gesundheit und Verbraucherschutz) Hamburg (animal protocol N008/2020; N111/2020). Gpr41/43 dKO mice kindly provided by Stefan Offermanns, Max-Planck-Institut für Herz- und Lungenforschung, Bad Nauheim, Germany (38) and wild type (WT) control littermates were bred and housed in the animal facility of the UKE at 22°C under a day-night cycle of 12 h and ad libitum access to drinking water and standard laboratory chow diet (P1324, Altromin, Germany) unless otherwise indicated. For our study, we analyzed female and male Gpr41/43 dKO and their WT littermates as controls as indicated. In another experimental set up, 13 weeks old Gpr41/43 dKO and WT mice were fed either control diet (EF D12450H, Ssniff, Germany) or 10% inulin-containing diet (EF E15744-34, Ssniff, Germany) for 6 weeks. Body composition analysis (fat mass and lean mass) was performed at the end of all experiments by using EchoMRI Analyzer (Zinsser-Analytic, Germany). At the end of the experiments, after a 4 h fasting period, portal and systemic ETDA blood was collected, organs were harvested, and samples for RNA isolation were snap-frozen in liquid nitrogen and stored at -80°C for further analysis. For bone histology, the dissected skeletons were fixed in 3.7% PBS-buffered formaldehyde for 18 h, before they were stored in 80% ethanol for further processing.

### 2.2 Oral glucose tolerance test

For oral glucose tolerance test, male WT and Gpr41/43 dKO mice received orally a glucose gavage (2 g/kg body weight). At indicated time points, glucose concentrations were determined in blood from tail vein using commercially available AccuCheck Aviva glucose sticks (Roche, Germany).

### 2.3 Indirect calorimetry

Energy expenditure was monitored through indirect calorimetry using Promethion Core systems (Sable Systems International, Las Vegas, USA) for 2 consecutive days. Weight matched 15-19 week old Gpr43/41 dKO mice and their wild type littermates were singly housed in Promethion control cabinets. Using this system, different parameters such as oxygen (O_2_) consumption (ml/min), carbon dioxide (CO_2_) production (mL/min), respiratory exchange ratio (RER = VCO_2_/VO_2_) and food intake (g) were recorded. Measurements were taken at 5 min intervals.

### 2.4 Skeletal phenotyping

For microCT analysis (µCT), one femur of each WT and Gpr41/43 dKO mouse was extracted and excess soft tissue removed. µCT scanning and evaluation was performed as previously described (39). In brief, femurs were scanned using a µCT 40 desktop cone-beam microCt (SCANCO Medical AG, Switzerland) with a voxel size of 10 µm (1000 projections per slice with 2048 samples and 200 s sample time at a tube energy of 55 kVp with an intensity of 145 mA) according to standard guidelines (40). Using the SCANCO µCT software suite, reconstructed slices were evaluated. Trabecular bone was analyzed in the distal metaphysis in a volume situated 2500 µm to 500 µm proximal of the distal growth plate. Cortical bone was analyzed in a 1000 µm long volume situated in the middle of the diaphysis. For bone histology, the lumbar vertebral bodies L1-L4, one tibia and one femur of each WT and Gpr41/43 dKO mouse were dehydrated in ascending alcohol concentrations and then embedded in methylmetacrylate for undecalcified histology as described in (41). Sections of 5 µm thickness were cut in the sagittal plane on a Microtec rotation microtome (Techno-Med GmbH, Germany) and stained by von Kossa/van Gieson and Toluidine blue staining procedures (42). Quantitative analysis of adipocytes was performed on toluidine blue stained proximal tibia and lumbar vertebral sections using OsteoMeasure histomorphometry system (OsteoMetrics, Decatur, GA, USA) (17). Histomorphometry was performed according to the ASBMR guidelines using the OsteoMeasure histomorphometry system (OsteoMetrics, Decatur, GA, USA) (43).

### 2.5 Bone turnover markers

To investigate burn turnover markers in plasma, EDTA blood was collected from WT and Gpr41/43 dKO mice at 6, 13 and 30 weeks of age, centrifuged at 10,000 x g for 5 min for plasma collection. Bone turnover markers were determined using procollagen type I N-terminal propeptide (PINP) ELISA (Cloud-Clone Corp, USA and RatLaps™ CTX-I EIA C-terminal telopeptide (CTX) (Immunodiagnostic Systems GmbH, Germany.

### 2.6 Cell culture

#### 2.6.1 Primary osteoblasts

To analyze the effect of SCFA acetate on primary murine osteoblasts, bone marrow cells were collected by centrifugation from long bones of 9 weeks old WT and Grp41/43 dKO mice. The cells were then plated in 24-well plates at a density of 3x10^6^ cells/well in α-MEM (Sigma-Aldrich, USA) supplemented with 10% (v/v) FBS (American Type Culture Collection, USA) and 100 U/ml penicillin/streptomycin (Life Technologies, USA) until they reached 80% confluency (day 0). To induce osteogenic differentiation, 50 μg/ml ascorbic acid (Sigma-Aldrich, USA) and 10 mM β-glycerophosphate (Sigma-Aldrich, USA) to the culture medium were added in the absence or presence of acetate (500 µmol) (Sigma-Aldrich, USA) in the cultures for 5 days. Alizarin red staining and quantification of mineralization were performed as described previously (39, 44).

#### 2.6.2 Primary osteoclasts

For primary osteoclast cultures, bone marrow cells were isolated from the tibiae and femora of 8-18-week-old C57Bl/6J mice. Cells were cultured in a-MEM (Sigma-Aldrich Corp.) supplemented with 10% FCS (Thermo Fisher Scientific Inc., Waltham, MA, USA), 100 U/ml penicillin/streptomycin (Life Technologies, Carlsbad, CA, USA) and 10 nM 1,25(OH)2 vitamin D3 (Sigma-Aldrich Corp.) at 37°C, 5% CO2 and 95% relative humidity. Medium was changed every other day and from day five of the culture onwards, 20 ng/ml M-CSF (PeproTech Inc., Rocky Hill, NJ, USA) and 40 ng/ml msRANKL (PeproTech Inc.) were added to the medium. After 12 days of differentiation, osteoclasts were stained for activity of tartrate-resistant acid phosphatase (TRAP) according to standard protocols. In brief, cells were fixed for 5 min with icecold methanol (Merck) for immediate TRAP staining. Cell were washed with water, dried for 2 min at room temperature, and developed with freshly prepared staining solution (40mmol·L-1 sodium acetate, Merck; 10 mmol·L-1 sodium tartrate, Merck; 0.1 mg·mL-1 naphtol AS-MX phosphate, Sigma; 0.6 mg·mL-1 Fast Red Violet LB salt, Sigma; 1% V/V N,N-Dimethylformamide, Sigma) (45). At the same time point, cultures with identical treatment were used for RNA extraction as described below.

#### 2.6.3 Primary chondrocytes

Primary chondrocytes were isolated from rib cage cartilage of 10-day-old C57Bl/6J mice by collagenase (Sigma-Aldrich; C9891) digestion in DMEM:F12 and subsequently cultured in DMEM:F12 supplemented with 10% FCS and 50 μg/ml ascorbic acid at 37°C, 5% CO2, 5% O2 and 95% relative humidity.

### 2.7 SCFAs analysis

Determination of SCFAs in portal blood was performed as described recently (28, 46). In brief, 30 µl of portal plasma was extracted in 293.75 µl ethanol added with 6.25 µl of internal standard mix (deuterated acetic acid/propionic acid/butyric acid). After vortexing and centrifugation, the supernatant was transferred into a fresh tube and 5 µl of 0.8 M NaOH solved in ethanol was added followed by evaporation of solvents using a vacuum centrifuge. The residual salts were dissolved in 50 µl EtOH and acidified with 10 µl 0.6 M succinic acid. GC-MS analysis of SCFA were performed by a TRACE 1310 gas chromatograph/ISQ 7000 mass selective detector (ThermoFisher Scientific, Dreieich, Germany) equipped with a Nukol Fused Silica Capillary Column (15 m x 0.32 mm x 0.25 µm film thickness) (Supelco/Sigma Aldrich, St. Louis, MO, USA). Peaks were identified by comparison of retention times and peak areas to standard chromatograms.

### 2.8 Gene expression analysis

Murine tissues and cultured osteoblasts were disrupted in TriFast (Peqlab) and using a TissueLyzser (Qiagen, Hilden, Germany). Nucleic acids were extracted with chloroform, and total RNA was isolated using the NucleoSpin RNA II kit (Macherey & Nagel, Düren, Germany) according to the manufacturer’s protocol using standard procedures (45). Genome-wide expression analysis was applied by using a Clariom D array kit (Thermo Fisher Scientific, Inc) as described recently (47) Three WT control samples were pooled and three acetate treated WT samples were pooled and prepared according to the manufacturer’s GeneChip™WT PLUS reagent kit manual. For Gene Chip hybridization, 5.5 μg of fragmented and labeled cDNA was incubated in hybridization solution at 45°C for 16 hours, before the Gene Chips (Clariom D, mouse) were washed using the Affymetrix Fluidics Station 450 (Affymetrix, Santa Clara,CA, USA). Microarrays were scanned with the Affymetrix Gene Chip Scanner 7G, and the signals were analyzed with the Transcriptome Analysis Console software (TAC 4.0; Thermo Fisher Scientific) using default analysis settings (version1) and Gene + Exon– signal space transformation–robust multiarray analysis (SST-RMA) as summarization (47).

Complementary DNA (cDNA) was synthesized from 400 ng of RNA using a High-Capacity cDNA Reverse Transcription kit (Applied Biosystems) according to the manufacturer’s protocol. Quantitative real-time PCR was performed with a QuantStudio™ 5 Real-Time PCR System (Thermo Fisher Scientific) using assays-on-demand primer (Applied Biosystems, assay IDs: *Acp5: Mm00475698_m1, Clcn7: Mm00442400_m1, Ctsk: Mm00484039_m1, Col1a1*: Mm00801666_g1, *Adipoq*: Mm00456425_m1, *Eno3*: Mm00468267_m1, *Gpr41*: Mm02621638_s1, *Gpr43*: Mm02620654_s1, *Kcnk1*: Mm00492791_m1, *Pparg*: Mm00440945_m1, *Phex*: Mm00448119_m1, *Ptgs2*: Mm00478374_m1, *Lpl*: Mm00434764_m1) that were premixed with TaqMan Universal MasterMix II (Applied Biosystems). Relative expression of genes of interest was calculated by normalization to housekeeper ribosomal protein lateral stalk subunit P0 (*Rplp0:* Mm01974474_gH or *Gapdh*: Mm99999915_g) mRNA using the 2^-ΔΔCt^ method.

For mRNA sequencing (RNAseq), total RNA was sequenced on a NovaSeq 6000 PE150 platform (Novogene). Bioinformatics analysis included mapping to the mouse reference genome, gene expression quantification, differential expression analysis as well as KEGG enrichment analysis of differentially expressed genes. For Volcano plot presentation, genes with an adjusted p-value < 0.01 and |log2(FoldChange)| > 0 were considered as differentially expressed. Classification of KEGG databases with padj < 0.05 were considered as significant enrichment.

### 2.9 Statistical analysis

All data present in the manuscript are presented as means ± standard error of the mean (SEM). Group sizes are indicated in the figure legends. For the comparison of two groups, statistical analysis was performed using unpaired two-tailed Student’s t test. For the comparison of multiple groups, we used two-way ANOVA with Tukey’s multiple comparisons test as indicated in the figure legends. GraphPad Prism 10.0.3 (GraphPad Software, Inc., La Jolla, CA, USA) was used for statistical calculations and p values below 0.05 were considered statistically significant.

## 3 Results

### 3.1 Expression pattern of Gpr41 and Gpr43 and metabolic phenotype of Gpr41 and Gpr43 deficiency

**Figure 1:**
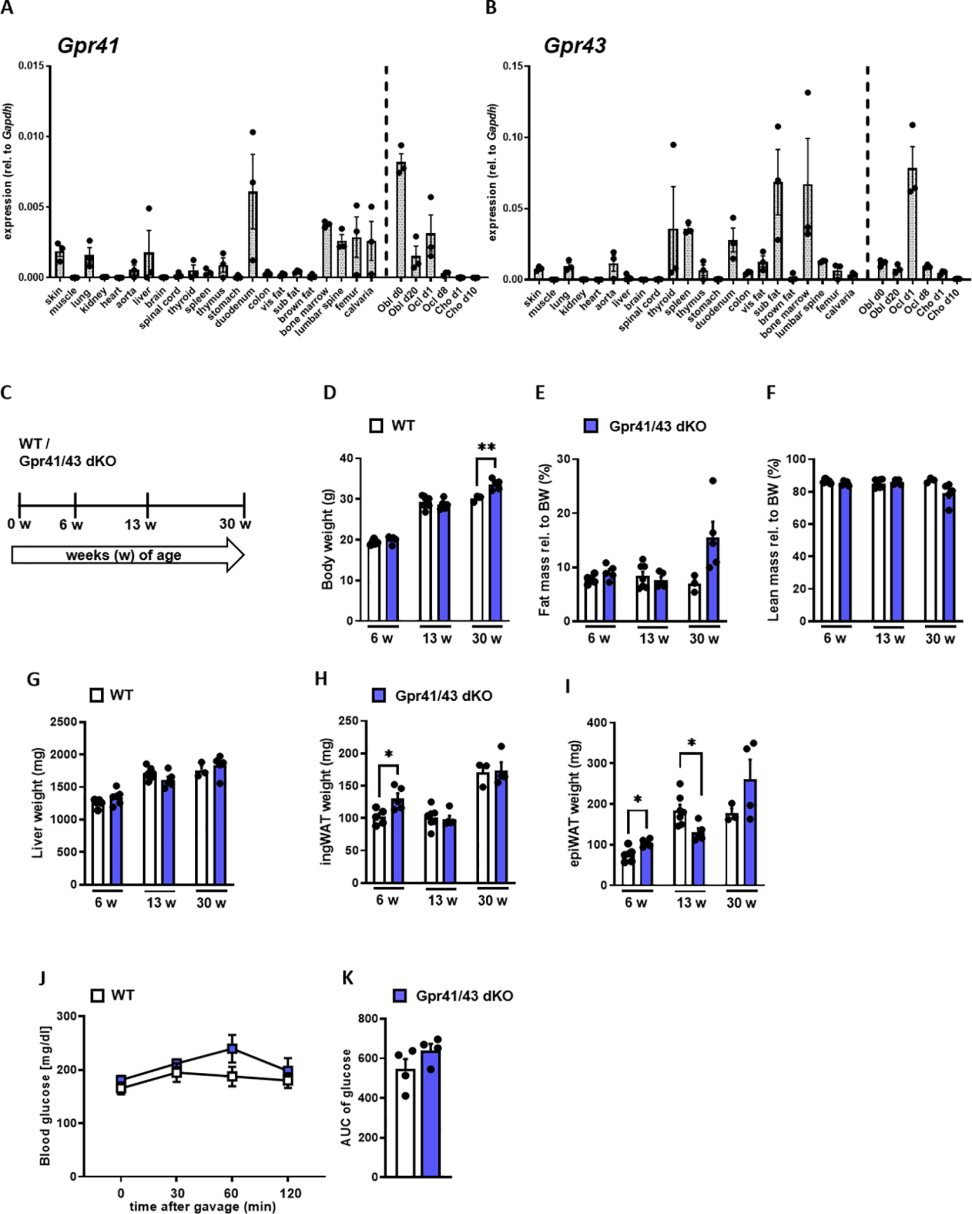
Expression analysis of *Gpr41* and *Gpr43* and body composition of Gpr41/43 dKO mice at different time points of age. **(A)** qRT-PCR expression analysis of *Gpr41* and **(B)** *Gpr43* in indicated tissues and primary osteoblasts, osteoclasts and chondrocytes at different differentiation time points relative to the respective *Gapdh* expression. n= 3 samples per tissue from 10 weeks old female C57Bl/6J. WT (white bars) and Gpr41/43 dKO (blue bars) mice were used to investigate body composition and organ weights at 6 (n= 5 WT, n=5 Grp41/43 dKO), 13 (n=7 WT, n=5 Gpr41/43 dKO) and 30 (n=3 WT, n=4-5 Gpr41/43 dKO) weeks of age. **(C)** Study design: WT and Gpr41/43 dKO mice were investigated at 6, 13 and 30 weeks of age and read outs were **(D)** body weight, **(E)** fat mass rel. to body weight (BW), **(F)** lean mass rel. to BW, **(G)** liver weight, **(H)** inguinal white adipose tissue (ingWAT), **(I)** epididymal white adipose tissue (epiWAT). **(J)** At indicated time points after gavage, glucose concentrations and **(K)** glucose quantification (area under the curve (AUC)) were determined in blood from tail vein of WT (n=4) and Gpr41/43 dKO (n=4) mice. Data were shown as dot plots with median values indicated as horizontal bars ± SEM analyzed by Student’s t-test. *p < 0.05 vs. Gpr41/43 dKO, **p < 0.01 vs. Gpr41/43 dKO.

In the first set of experiments, we compared the expression pattern of *Gpr41* and *Gpr43* in different murine tissues and non-differentiated as well as differentiated primary murine bone cells of C57Bl6/J wild type mice (**Figures 1A, B**). The expression levels of *Gpr43* were generally higher compared to expression levels of *Gpr41*. Intriguingly, *Gpr43* exhibits at least 17-fold higher expression than *Gpr41* in the bone marrow and a 70-fold higher expression in subcutaneous adipose tissue compared to *Gpr41* for the same tissue. In line with the established role in enterocytes (24, 48), we determined high level of *Gpr41* and *Gpr43* in the duodenum of mice. As expected, we observed the highest levels of Gpr41 in the duodenum of mice, consistent with its important function in the gut, as SCFAs are produced by bacterial fermentation and activate Gpr41 and Gpr43 on enterocytes (24, 25). Notably, the second highest expression of *Gpr41* was detected in bone tissues such as bone marrow, femur, calvaria and lumbar spine. Moreover, *Gpr41* was expressed by osteoblasts and osteoclasts at various differentiation time points but not by chondrocytes. In this comparison the *Gpr41* expression appears to be more relevant, as the expression in skeletal tissues and osteoblasts is apparently higher than in most other organs. Given the expression pattern for these SCFA receptors, we first studied the metabolic phenotype of a recently described mouse model lacking both *Gpr41* and *Gpr43* (38). For this purpose, we first compared body weight, lean and fat mass, organ weights of 6, 13 and 30 weeks old wild type and Gpr41/43 double KO mice (**Figures 1C–I**). Deletion of *Gpr41/43* was associated with a slightly higher body weight only at 30 weeks of age (**Figure 1D**), whereas no differences were observed with regard to fat mass (**Figure 1E**), lean mass (**Figure 1F**), weights of liver (**Figure 1G**) or inguinal white adipose tissue (ingWAT) (**Figure 1H**). The weight of epididymal white adipose tissue (epiWAT) slightly differed between WT and Gpr41/43 dKO mice at 6 and 13 weeks of age (**Figure 1I**). Comparison of blood glucose during an oral glucose tolerance revealed no differences between WT and Gpr41/43 dKO mice (**Figures 1J, K**). Furthermore, energy expenditure, respiratory exchange ratio, oxygen consumption and food intake were similar when comparing *Gpr*43/41 dKO and their wild type littermates (**Supplementary Figures 1A–E**). In the same way, Gpr41/43 did not affect body and organ weights in female mice (**Supplementary Figures 2A–F**).

### 3.2 Impact of *Gpr41* and *Gpr43* deficiency on bone mass *in vivo*

**Figure 2:**
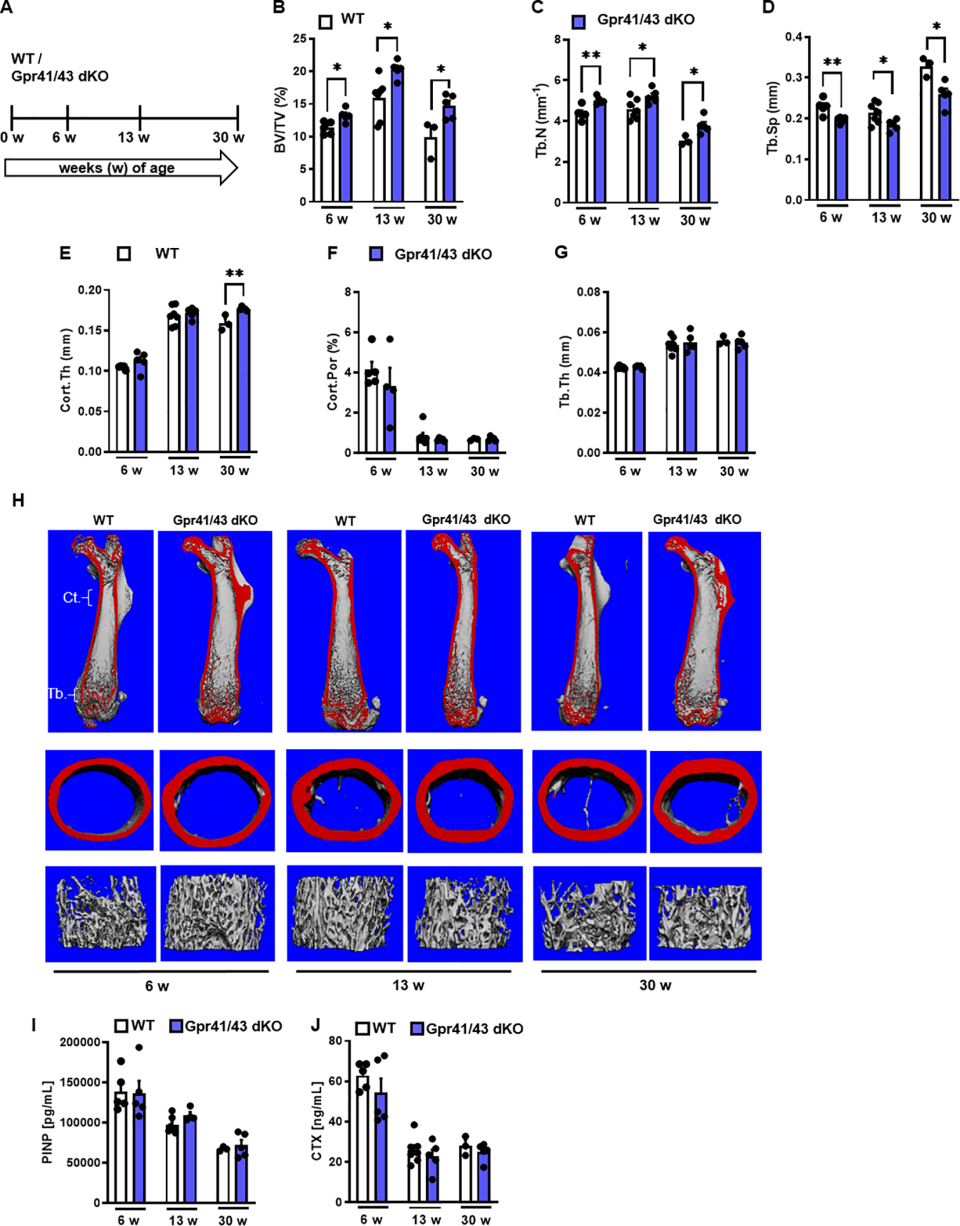
Skeletal phenotype of Gpr41/43 double-deficient mice. **(A)** Study design: Femoral phenotype of WT (white bars) and Gpr41/43 dKO (blue bars) mice were investigated at 6 (n=5 WT, n=5 Grp41/43 dKO), 13 (n=7 WT, n=5 Gpr41/43 dKO), and 30 (n=3 WT, n=4-5 Gpr41/43 dKO) weeks of age. µCT-based quantification of **(B)** trabecular bone volume per tissue volume (BV/TV), **(C)** trabecular numbers (Tb.N), **(D)** trabecular spacing (Tb.Sp), **(E)** cortical thickness (Cort.Th), **(F)** cortical porosity (Cort.Por) and **(G)** trabecular thickness (Tb.Th). **(H)** Representative µCT images of femora from 6 13 and 30 weeks old male WT mice showing whole femurs (top, the virtual cut plane appears red), cortical (Ct., middle) and trabecular bone (Tb., bottom). **(I)** Bone formation marker procollagen type 1 N-terminal propeptide (PINP) and **(J)** bone turnover marker C-terminal telopeptide (CTX) were measured in blood plasma at indicated time points of age. Data were shown as dot plots with median values indicated as horizontal bars ± SEM analyzed by Student’s t-test. *p < 0.05 vs. Gpr41/43 dKO, **p < 0.01 vs. Gpr41/43 dKO.

**Figure 3:**
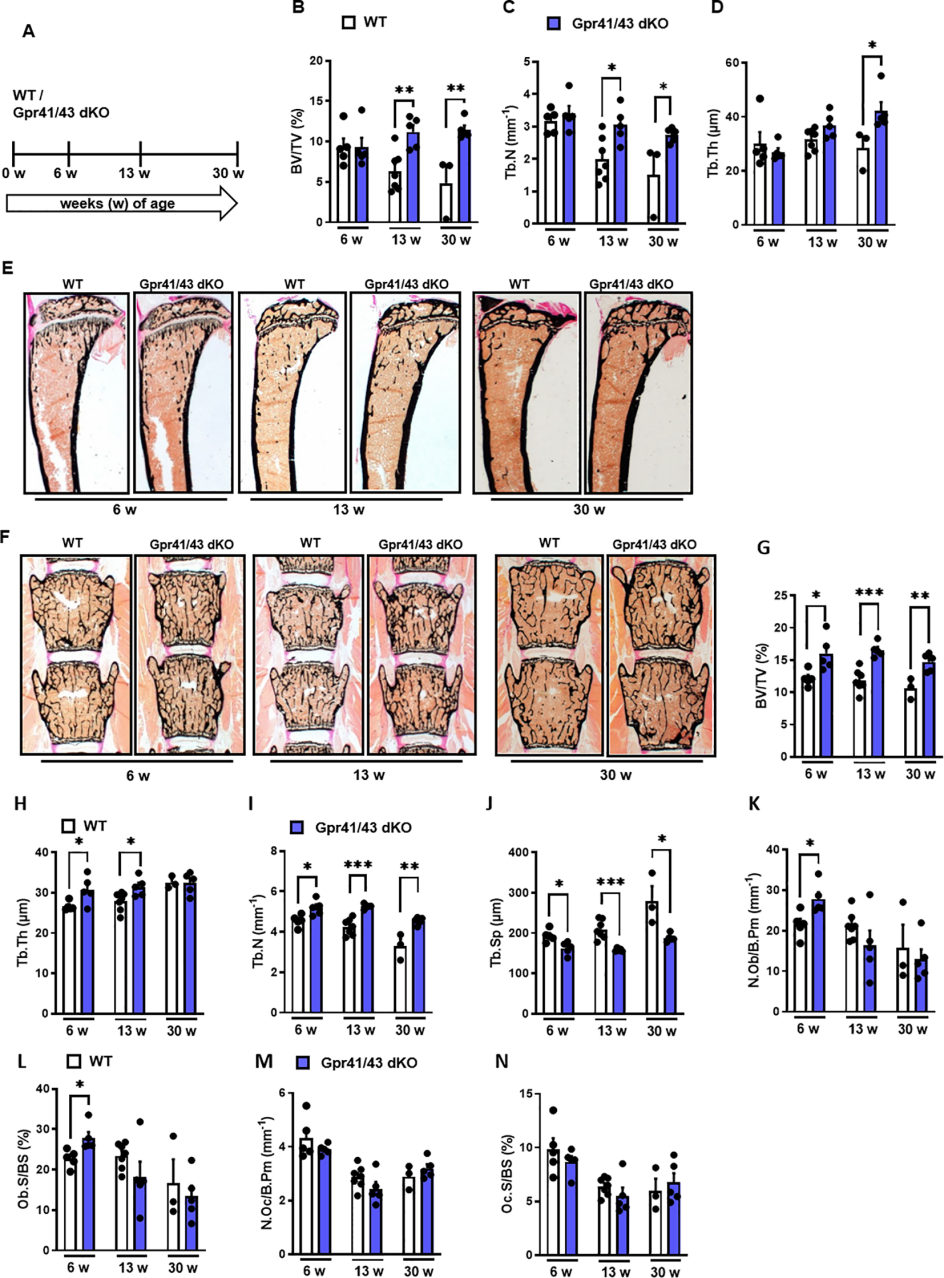
Deletion of Gpr41 and Gpr43 increases trabecular bone mass in mice. **(A)** Study design of histomorphometric analysis from WT (white bars) and Gpr41/43 dKO (blue bars) mice at 6, 13 and 30 weeks of age. **(B-D)** Histomorphometric evaluation of trabecular bone parameters in tibia. **(B)** Bone volume per tissue volume, **(C)** trabecular numbers (Tb.N), **(D)** trabecular thickness (Tb.Th). Data were shown as mean values ± SEM and analyzed by Student’s t-test. n=3-7 mice per group. *p < 0.05 vs. Gpr41/43 dKO, **p < 0.01 vs. Gpr41/43 dKO. **(E)** Representative undecalcified histological sections of Von Kossa/van Gieson stained tibia from 6, 13 and 30 weeks old male WT and Gpr41/43 dKO. **(F)** Representative undecalcified histological sections of vertebral bodies from 6, 13 and 30 weeks old male WT and Gpr41/43 dKO mice. Von Kossa/van Gieson stain. **(G-N)** Histomorphometric evaluation of trabecular bone parameters in the same sections. **(G)** Bone volume per tissue volume, **(H)** trabecular thickness (Tb.Th), **(I)** trabecular numbers (Tb.N) and **(J)** trabecular spacing (Tb.Sp). **(K-N)** Histomorphometric evaluation of cellular bone remodeling parameters in the same sections of vertebral bodies. **(K)** Number of osteoblasts/bone perimeter (N.Ob/B.Pm), **(L)** osteoblasts surface per bone surface (Ob.S/BS), **(M)** number of osteoclasts/bone perimeter (N.Oc/B.Pm), **(N)** osteoclasts surface per bone surface (Oc.S/BS). Data were shown as dot plots with median values indicated as horizontal bars ± SEM and analyzed by Student’s t-test. n=3-7 mice per group. *p < 0.05 vs. Gpr41/43 dKO, **p < 0.01 vs. Gpr41/43 dKO, ***p < 0.001 vs. Gpr41/43 dKO.

Next, we compared the skeletal phenotype between male WT and Gpr41/43 dKO mice at 6, 13 and 30 weeks of age by microCT scanning (**Figures 2A–H**). Analyses of femoral bones indicated an age-dependent increase of trabecular bone volume (**Figure 2B**) and trabecular numbers (**Figure 2C**) in Gpr41/43 dKO compared to wild type controls, which is in line with reduced trabecular spacing (**Figure 2D**). The thickness of the femoral cortex was only significantly increased at later age in the Gpr41/43 dKO mice compared to the control mice (**Figure 2E**). Cortical porosity (**Figure 2F**) and trabecular thickness (**Figure 2G**) did not differ between WT and Gpr41/43 dKO. Bone formation marker PINP and bone turnover marker CTX did not identify significant genotype-dependent differences (**Figures 2I, J**). In female Gpr41/43 dKO mice, we found a similar bone phenotype at 31 weeks of age compared to WT controls (**Supplementary Figures 3A–G**). Already here it is important to note that increased bone mass was associated with lower adipocyte number and bone marrow adiposity in Gpr41/43 dKO mice (**Supplementary Figures 5A, B**). To further characterize the skeletal phenotype, we investigated tibia (**Figures 3A–E**) and lumbar spine (**Figures 3F–N**) by histological and histomorphometric quantification analysis. Deletion of Gpr41/43 resulted in significant increase in bone volume to tissue volume ratio (**Figure 3B**) and higher trabecular numbers at 13 and 30 weeks of age (**Figure 3C**). Trabecular thickness in tibia was only affected by Gpr41/43 deficiency at 30 weeks of age (**Figure 3D**). Histomorphometric evaluation of trabecular bone parameters of lumbar spine (**Figures 3F–N**) displayed a significant gain in bone mass of Gpr41/43 dKO versus wild type controls, as shown by increased trabecular bone volume (**Figure 3G**), trabecular thickness (**Figure 3H**), trabecular numbers (**Figure 3I**), and decreased trabecular spacing (**Figure 3J**). Similar to males, histological analysis of bone mass and trabecular numbers in female spine (**Supplementary Figures 3H–K**) and femora (**Supplementary Figures 3L–O**) were significantly higher in Gpr41/43 dKO mice compared to WT controls. Deficiency of Gpr41/43 led to decreased trabecular spacing in femora of female mice (**Supplementary Figure 3P**). Histomorphometric evaluation of cellular bone remodeling parameters in lumbar spine showed an increase in numbers of osteoblasts per bone perimeter (**Figure 3K**) and osteoblast surface per bone surface (**Figure 3L**) of Gpr41/43 dKO mice compared to WT male mice, while this phenotype was not accompanied by changes in numbers of osteoclast parameters at any time point (**Figures 3M, N**). Taken together, deletion of SCFA receptors Gpr41 and Gpr43 increase bone mass, an effect that is most likely mediated by an increased number of bone-forming osteoblasts.

### 3.3 Effect of inulin feeding on bone remodeling in Gpr41/43 dKO mice

**Figure 4:**
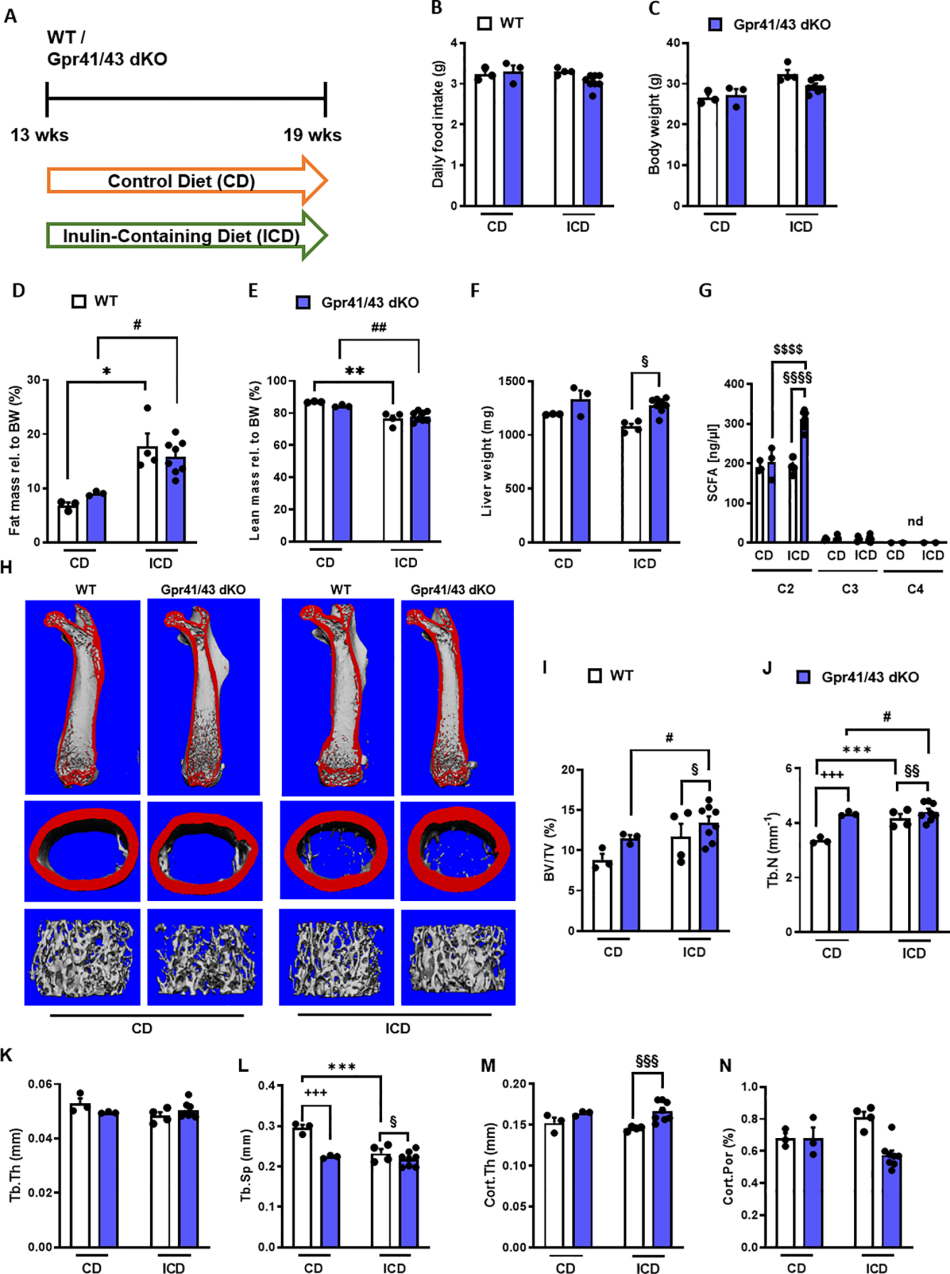
Effect of inulin-containing on bone mass in Gpr41/43-defient mice. **(A)** Study design: 13 weeks old male WT (white bars) and Gpr41/43 dKO mice (blue bars) were fed an inulin-containing diet (ICD) or a respective control diet (CD) for 6 weeks. **(B)** Average daily food intake, **(C)** body weight, **(D)** fat mass relative to body weight (BW), **(E)** lean mass relative to BW, **(F)** liver weight. **(G)** By using GC-MS analysis, levels of SCFAs in portal plasma were analyzed acetate (C2), propionate (C3), butyrate (C4), nd (not defined). **(H)** Representative µCT images of femora from WT and Gpr41/43 dKO showing whole femurs (top, the virtual cut plane appears red), cortical (Ct., middle) and trabecular bone (Tb., bottom). µCT-based quantification of **(I)** trabecular bone volume per tissue volume (BV/TV), **(J)** trabecular numbers (Tb.N), **(K)** trabecular thickness (Tb.Th), **(L)** trabecular spacing (Tb.Sp) **(M)** cortical thickness (Cort.Th), **(N)** cortical porosity (Cort.Por), **(N)**,. Data were shown as dot plots with median values indicated as horizontal bars ± SEM. ^+++^p < 0.001 WT CD vs. Gpr41/43 dKO CD,*p < 0.05 WT CD vs. WT ICD, **p < 0.01 WT CD vs. WT ICD, ***p < 0.001 WT CD vs. WT ICD, ^#^p < 0.05 Gpr41/43 dKO CD vs Gpr41/43 dKO ICD, ^##^p < 0.01 Gpr41/43 dKO CD vs Gpr41/43 dKO ICD, ^§^p < 0.05 WT ICD vs Gpr41/43 dKO ICD, ^§§^p < 0.01 WT ICD vs Gpr41/43 dKO ICD, ^§§§^p < 0.01 WT ICD vs Gpr41/43 dKO ICD determined by two-way ANOVA. WT, CD: n=3, Gpr41/43, CD: n=3, WT, ICD: n=4, Gpr41/43 dKO, ICD: n=8.

Fermentation of dietary fibers such as the fructan inulin by gut microbes results in the production of SCFAs (49), and it has been reported that feeding inulin has a positive osteoanabolic effect and increases bone mineralization (50–52). However, whether the described effects of fiber-rich diets and/or SCFAs on bone are mediated by *Gpr41* and *Gpr43* remains unclear. To investigate whether the improved bone mass phenotype caused dietary inulin supplementation is blunted in Gpr41/43 dKO mice, 13 weeks old WT and Gpr41/43 dKO were fed a chow control diet (CD) or inulin-containing diet (ICD) for 6 weeks (**Figure 4A**). No differences were observed when comparing the food intake of these groups (**Figure 4B**) and their body weight (**Figure 4C**). Interestingly and despite the unaltered body weights, ICD feeding caused a genotype-independent increase in fat mass (**Figure 4D**) and a concomitant decrease in lean mass (**Figure 4E**). While we observed a trend in the CD-fed mice, inulin supplementation resulted in a higher liver weight in Gpr41/43 dKO mice compared to control mice (**Figure 4F**), suggesting that SCFA regulate hepatocellular mass or hepatic lipid accumulation via Gpr41/43 signaling. To study the effect of inulin feeding on SCFA levels, we determined acetate (C2), propionate (C3) and butyrate (C4) levels in CD- and ICD-fed mice (**Figure 4G**). It is of note that in response to ICD feeding acetate levels were higher only in Gpr41/43 dKO mice, and that acetate has a much higher concentration compared to propionate and butyrate in the portal circulation, indicating the high clearance of propionate and butyrate by intestinal cells and hepatocytes. Furthermore, these data suggest that only concentration of acetate would be sufficient to agonize *Gpr41* and/or *Gpr43* in peripheral tissues such as bone or adipose tissue.

**Figure 5:**
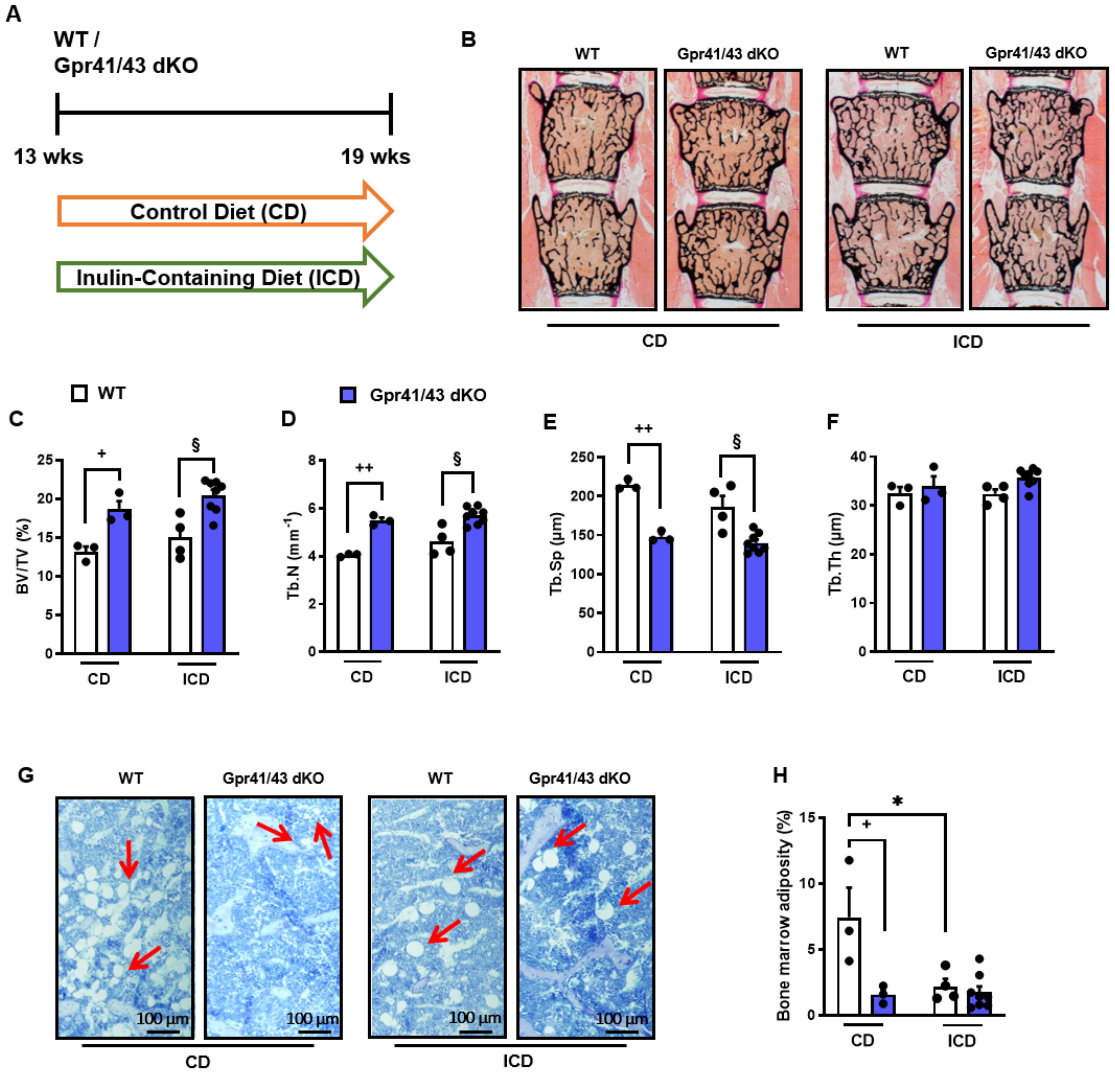
Inulin-containing diet decreases bone marrow adiposity. **(A)** Study design: 13 weeks old male WT (white bars) and Gpr41/43 dKO mice (blue bars) were fed an inulin-containing diet (ICD) or a respective control diet (CD) for 6 weeks. **(B)** Representative undecalcified histological sections and of vertebral bodies from 19 weeks old male WT and Gpr41/43 dKO mice fed an inulin-containing diet (ICD) or a respective control diet (CD). **(C–F)** Histomorphometric evaluation of trabecular bone parameters in the same sections. **(C)** Bone volume per tissue volume, **(D)** trabecular numbers (Tb.N), **(E)** trabecular spacing (Tb.Sp), **(F)** trabecular thickness (Tb.Th). **(G)** Representative images of toluidine blue stained tibia sections and red arrows indicate bone marrow adipocytes. **(H)** Quantification of bone marrow adiposity from the same section. Data were shown as dot plots with median values indicated as horizontal bars ± SEM ^+^p < 0.05 WT CD vs. Gpr41/43 dKO CD, ^++^p < 0.01 WT CD vs. Gpr41/43 dKO CD, *p < 0.05 WT CD vs. WT ICD, ^#^p < 0.05 Gpr41/43 dKO CD vs. Gpr41/43 dKO ICD, ^§^p < 0.05 WT ICD vs. Gpr41/43 dKO ICD, determined by two-way ANOVA. WT, CD: n=3, Gpr41/43, CD: n=3, WT, ICD: n=4, Gpr41/43 dKO, ICD: n=8.

To assess whether feeding inulin alters bone remodeling via Gpr41/43, we next studied the microarchitecture of femoral bones (**Figures 4H–N**). Inulin supplementation significantly enhanced bone mass in Gpr41/43 dKO mice as determined by the BV/TV ratio, whereas only a trend was observed for this parameter in WT mice (**Figure 4I**). Trabecular numbers were increased by feeding inulin in both genotypes, whereas Gpr41/43 dKO have higher values in comparison to controls (**Figure 4J**). The average trabecular thickness (**Figure 4N**) did not differ between WT and Gpr41/43 dKO fed control or inulin diet. In line with this, inulin diet significantly reduced trabecular spacing in WT mice (**Figure 4L**). In contrast, the cortical bone was unaffected by this treatment as determined by cortical thickness (**Figure 4M**), cortical porosity (**Figure 4N**). In lumbar spine of WT and Gpr41/43 dKO mice, inulin feeding did not cause an increase in bone mass, as determined by histomorphometry of lumbar spine sections (**Figures 5A–F**). However, it is of note that on both diets an increased bone mass was detected in Gpr41/43 dKO compared to WT mice. Overall, it can be summarized that the osteoanabolic effect observed in Gpr41/43 deficient mice is independent of feeding a fiber-rich diet. Moreover, histomorphometric analysis indicates that inulin feeding significantly reduces bone marrow adiposity in WT mice (**Figures 5G, H**), which is in line with a beneficial effect of inulin on bone health (49, 53). Compared to WT controls, Gpr41/43 dKO mice display lower bone marrow adiposity already under chow diet conditions (**Supplementary Figures 4A, B**), suggesting that Gpr41/43-dependent signaling could influence the differentiation of mesenchymal stem cells to osteoblasts versus adipocytes.

### 3.4 Impact of acetate and Gpr41/43-dependent signaling on osteogenic and adipogenic differentiation

**Figure 6:**
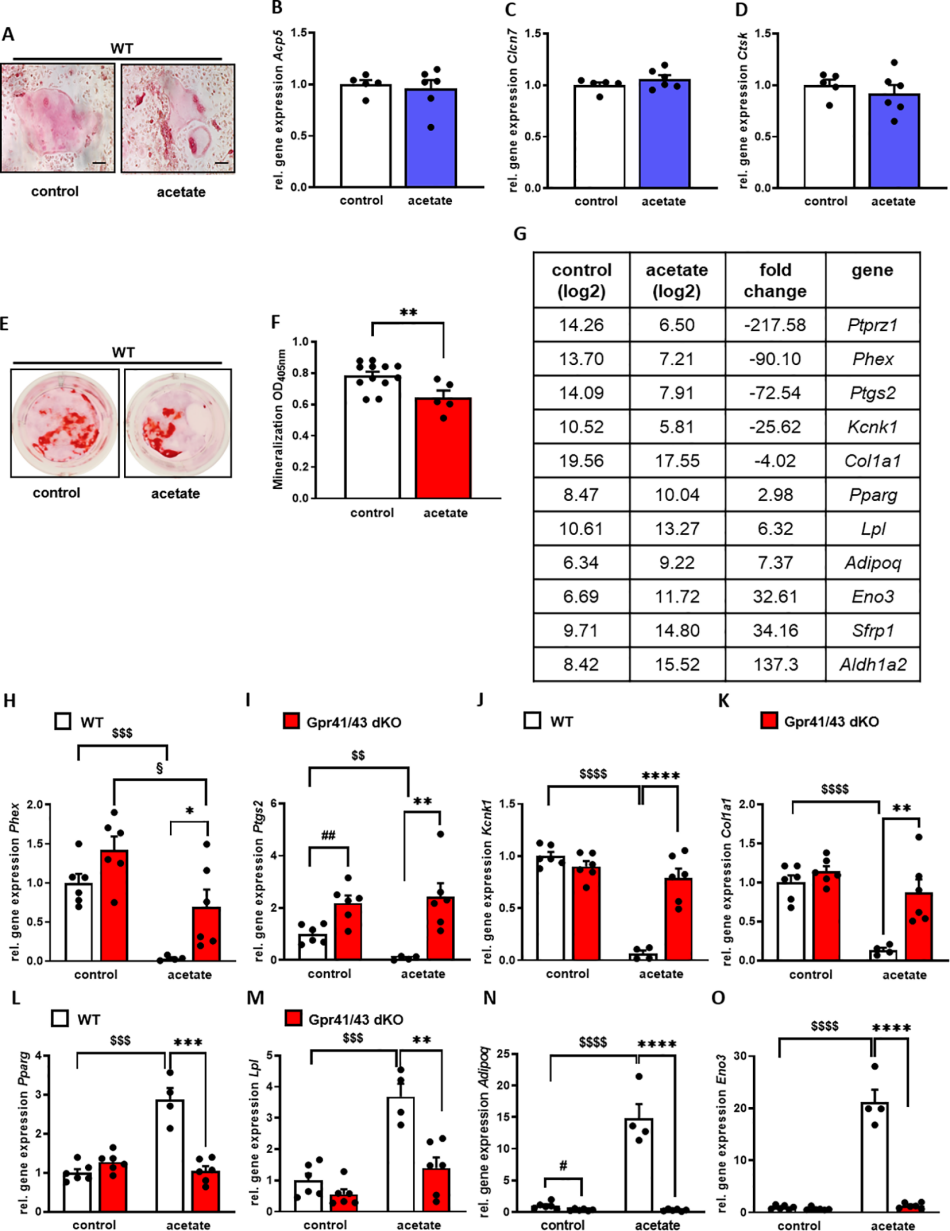
Acetate facilitates adipocytic differentiation of mesenchymal stem cells. **(A-D)** Bone marrow cells from tibiae and femora of 8-18-week-old C57Bl/6J mice were isolated and differentiated into osteoclasts. **(A)** After 12 days of differentiation, osteoclasts were stained for activity of tartrate-resistant acid phosphatase (TRAP). **(B-D)** Relative gene expression level (fold difference) of indicated osteoclast markers *Acp5*, *Clcn7* and *Ctsk* of WT (n=5 white bars) and Gpr41/43 dKO (n=6) bdlue bars) osteoclasts. Data are shown as dot plots with median values indicated as horizontal bars ± SEM. **(E–O)** Bone marrow from WT mice was isolated and mesenchymal stem cells were cultured with cultivation medium for 7 days followed by cultivation with osteoblastic medium in the absence or presence of acetate (500 µmol) for further 5 days. **(E)** Alizarin red staining of control (white bar) and acetate-treated WT osteoblasts (red bar). **(F)** Quantification of extracellular matrix mineralization from control and acetate-treated WT osteoblasts. **(G)** The expression pattern of downregulated and induced adipocyte and osteoblast marker genes, as determined by Gene Chip hybridization comparing control (n=3 pooled) and acetate-treated cells (n=3 pooled). *Gapdh* expression was monitored as positive loading control. Data are shown as dot plots with median values indicated as horizontal bars ± SEM. **p < 0.01 determined by Student’s t-test. Control WT osteoblasts n=12, acetate-treated WT osteoblasts n=5. **(H-O)** Relative gene expression level (fold difference) of indicated osteoblast markers of WT (white bars) and Gpr41/43 dKO mice osteoblasts (red bars) **(H-K)** *Phex*, *Ptgs2*, *Kcnk1*, *Col1a1* and **(L-O)** adipocyte markers *Pparg*, *Lpl*, *Adipoq*, *Eno3*. Data are shown as dot plots with median values indicated as horizontal bars ± SEM, and different icons indicate significant differences between groups (*p < 0.05, **p < 0.01, ***p < 0.001, ****p < 0.0001, ^$$^ p < 0.01, ^$$$^p < 0.001, ^$$$$^p< 0.0001, ^#^p < 0.05, ^##^ p < 0.01, ^§^p < 0.05) determined by two-way ANOVA. Control WT osteoblasts n=6, acetate-treated WT osteoblasts n=4. Control Gpr41/43 dKO osteoblasts n=6, acetate-treated Gpr41/43 dKO osteoblasts n=6.

A number of studies demonstrate a prominent role of SCFAs in white adipose tissue and pro- and anti-adipogenic functions of *Gpr41* and *Gpr43* have been reported (54). In order to investigate the functional significance of acetate, the most abundant short chain fatty acid in plasma, for osteogenic differentiation, we first isolated mesenchymal stem cells from the bone marrow of mice and differentiated them into osteoclasts and osteoblasts with and without the addition of acetate. We observed that osteoclast differentiation (**Figures 6A–D**) was not affected in the presence of acetate, unlike it was the case for osteoblast differentiation. In line with an anti-osteoanabolic effect, treatment of primary wild type osteoblasts with acetate for 5 days resulted in reduced extracellular matrix mineralization as determined by Alizarin red staining (**Figures 6E, F**). To study the underlying mechanisms, we performed an unbiased microarray RNA expression analysis. Among the 50 genes with the most pronounced differential expression (**Supplementary Table 1**), we found five known markers of osteoblastogenesis, *Ptprz1*, *Phex*, *Ptgs2*, *Kcnk1* and *Col1a1*, which were strongly downregulated by acetate (**Figure 6G**). It is noteworthy that even under conditions of osteogenic differentiation, acetate treatment resulted in a profound induction of typical adipogenic genes including *Pparg*, *Lpl*, *Adipoq*, *Eno3*, *Sfrp1*, and *Aldh1a2* (**Figure 6G**). To investigate whether these effects mediated by acetate are caused by Gpr41/43-dependent signaling, we studied gene expression of primary osteoblasts from WT and Gpr41/43-deficient mice after differentiation in the absence or presence of acetate (**Figures 6H–O**). By qPCR analysis, we confirmed that acetate treatment substantially decreased the osteoblastic markers *Phex*, *Ptgs2*, *Kcnk1* and *Col1a1* in WT osteoblasts (**Figures 6H–K**). These markers were significantly higher in Gpr41/43-deficient osteoblasts, and the anti-osteoanabolic effect of acetate was strongly attenuated or even blunted (**Figures 6H–K**). Conversely and in line with an adipogenic effect of acetate, the expression of *Pparg*, *Lpl*, *Adipoq and Eno3* were significantly higher in acetate-treated wild type adipocytes (**Figures 6L–O**). In line with lower bone marrow adiposity *in vivo* (see **Figures 5G, H**, **Supplementary Figures 4A, B**, **Supplementary Figures 5A–C**), the adipogenic effect of acetate was completely abrogated in differentiated Gpr41/43 cells (**Figures 6L–O**). To define the potential intracellular signaling pathway, which might be regulated by acetate-dependent activation of Gpr41/43, we performed RNAseq analysis of wild type and Gpr41/43-deficient osteoblasts treated without or with acetate (**Supplementary Figures 6**, **7**, **Supplementary Table 2**). KEGG analysis indicated that the differentially expressed genes (DEGs) were enriched in a number of pathways. Notably, the MAPK signaling pathway, the Wnt signaling pathway and TGF-beta signaling pathway, all known to be important for bone formation were regulated by acetate in wild type but not in Gpr41/43-deficient osteoblasts (55, 56). In addition, the direct comparison of wild type versus knockout osteoblasts suggests that parathyroid hormone receptor signaling is modulated (**Supplementary Figure 7**).

Overall, these data indicate that acetate-dependent signaling via Gpr41/43 favors an adipogenic differentiation fate of bone marrow mesenchymal stem cells, potentially explaining the higher bone mass of Gpr41/43-deficient mice.

## 4 Discussion

The gut microbiome plays an essential role in bone health and a disturbed gut microbiota negatively affects bone remodeling, and increases the osteoporosis and fracture risk (18, 19). The interplay between gut microbiota and bone cells are in part explained by SCFAs (30, 57), which are produced by the fermentation of non-digestible polysaccharides by bacterial enzymes present in the gut lumen (24, 25). Mechanistically, SCFAs can signal via ligation of the G protein-coupled receptors Gpr41 and 43. Recently, Montalvany-Antonucci et al. demonstrated a regulatory role of SCFAs as suppressors of bone resorption acting via Gpr43 on bone osteoclasts (58). Furthermore, Lucas et al. showed a beneficial effect of SCFAs on bone mass by feeding mice a high-fiber diet or by providing SCFAs-supplemented drinking water. In addition, they showed that propionate and butyrate act directly on murine osteoclasts by inhibition of osteoclasts differentiation (57). However, the potential role of Gpr41/Gpr43-dependent signaling for osteoblasts and bone mass remains to be elucidated. In the current study, we showed that both Gpr41 and Gpr43 are highly expressed in mouse bone tissues, osteoblasts and osteoclasts. Based on these results and the fact, that published data on bone health merely underlie either Gpr41 or Gpr43 deletion and focus more on osteoclasts (57–59), we performed bone analysis of Gpr41 and Gpr43 double knockout mice (38). Under normal dietary conditions, we did not observe differences in insulin levels (data not shown) or other metabolic parameters. Notably, we found that Gpr41/43 dKO mice showed an increased bone mass in the axial and appendicular skeleton. Moreover, numbers of osteoclasts were similar between Gpr41/43 dKO and their wild type littermates, indicating a negligible role of Gpr41/Gpr43 in bone resorption in our experimental setting. In line, the number of osteoblasts in lumbar spine of Gpr41/43 dKO mice were significantly higher at 6 weeks of age compared to WT cells suggesting a role of Gpr41/43 in bone formation. Next to bone cells, Gpr41 and Gpr43 expression has been shown in a various tissues, e.g. expression of Gpr41 was previously reported in white adipocytes, peripheral nerves, pancreatic β-cells, enteroendocrine cells, myeloid dendritic cells and the thymus (33, 60, 61). Gpr43 is expressed on cells of distal ileum, colon, and with highest expression detected in immune cells such as monocytes and neutrophils (60–62). Conflicting data exist in the literature about the expression of Gpr43 in white adipocytes (63, 64). Moreover, Zaibi et al. demonstrated that deletion of Gpr41 also reduced levels of Gpr43 expression (64). These various cell types could contribute directly or indirectly to the bone phenotype described here, as animals with global deficiency were examined for this study. Even if this represents a limitation, the *in vivo* results could be validated at the molecular level in primary osteoblasts. Binding of SCFAs to Gprs leads to an intracellular release of Ca^2+^ and to activation of different downstream signaling pathways, such ERK/MAPK, JNK, p38 or Akt/PI3K kinase cascades (65). To define the relevance of SCFAs for bone health via G-protein receptor signaling pathways, we investigated differentially expressed genes and performed KEGG pathway analysis in wild type and knockout osteoblasts. Notably, a number of intracellular signaling pathways important for bone formation, e.g. Wnt, MAPK and TGF-beta, are modulated by acetate in wild type but not in Gpr41/43-deficent osteoblasts. Future studies are needed to define how exactly the underlying intracellular signaling pathways are affected by these G-protein coupled receptors.

In the context of the current study it is of note that supplementation of fiber or SCFAs increases bone mass (57, 66). However, it is not clear, whether inulin supplementation can cause an increase in systemic SCFAs levels that are sufficient to activate Gpr41/43 signaling pathways on bone cells. In the current study, we found that the inulin-mediated increase in bone mass is largely independent of Gpr41/43, suggesting alternative processes how inulin promotes bone formation. For instance, it has been demonstrated that SCFAs promote calcium transport across colonic cells and subsequent bone mineralization after supplementation of fiber-rich diets (inulin-type fructans) (51, 67). Moreover, inulin can be fermented into metabolites other than SCFAs, such as lactate, hydrogen, methane or carbon dioxide (68), which could directly or indirectly modulate bone remodeling. However, we found that Gpr41/43 dKO mice display a high bone mass phenotype, and acetate-treated Gpr41/43-deficient osteoblasts are characterized by a higher expression of osteogenic markers than their wild type control cells. Together this indicates that systemic physiological acetate levels can inhibit osteoblast differentiation via Gpr41/43 signaling. Moreover, increased bone mass in Gpr41/43-deficient mice is inversely associated with lower abundance of lipids and lower bone marrow adiposity. Together with the abrogated effect of acetate on adipogenic expression, these date suggest that SCFA-dependent Gpr41/43 signaling have a decisive role for the differentiation fate of mesenchymal precursor cells within the bone marrow.

Osteoblast and adipocyte differentiation is closely related, as both cell types originate from common mesenchymal progenitors and the differentiation pathway between osteoblastogenesis and adipogenesis is influenced by various endocrine and paracrine factors (69–71). In addition, mature osteoblasts and adipocytes can dedifferentiate and transdifferentiate from one into the other cell type demonstrating the close relationship between osteoblasts and adipocytes (72). In contrast to many studies using supraphysiological concentrations of butyrate, we incubated osteoblasts in the absence or presence of physiological concentration of acetate, the SCFA with the by far highest levels in plasma (30). In this context, it is of note that in diabetic conditions that are characterized by elevated acetate concentration the lack of Gpr41/43 resulted in an impaired insulin secretion (37). Herein, we could show that acetate treatment of mesenchymal stem cells led to an increase in adipogenic differentiation and to reduced osteoblast differentiation, suggesting that acetate promotes adipocyte maturation. These effects were blunted in Gpr41/43^-/-^ cells, indicating that systemic metabolites such as the SCFA acetate might regulate bone formation by GPR-dependent signaling. The most abundant SCFA acetate acts as a substrate for hepatic lipogenesis and cholesterol biosynthesis (73, 74). In addition, to maintaining energy homeostasis, the metabolite acetate can also be generated by other body cells than enterocytes via conversion from acetyl-coA or acetaldehyde (75). In line with this, it was reported that acetate serves as a critical carbon source for lipid synthesis explaining our data and suggesting a more local effect of acetate which is utilized by mesenchymal stem cells in the bone marrow (76). Thus, this Gpr41/43-dependent effect determining the cellular fate could be particularly detrimental in individuals with diabetes. The higher levels of acetate in the blood described in this group of patients, who are at high risk of developing osteoporosis (38, 77–79), could trigger adipocyte differentiation and thus prevent osteoblast differentiation of mesenchymal stem cells via Gpr41/43. In mice, acetate activates Gpr41 and Gpr43 with similar potency (80), which was one of the reasons why we studied Gpr41/43 deficient mice. In humans, SCFAs bind with different affinity to Gpr41 (C3=C4=>C2) and Gpr43 (C2=C3>C4>) (60, 61, 81). Together with the results of the current study in mice, this suggests that Gpr43 antagonists may have osteoanabolic therapeutic potential, particularly in patient subgroups with osteoporosis and high systemic acetate levels.

**Figure 7:**
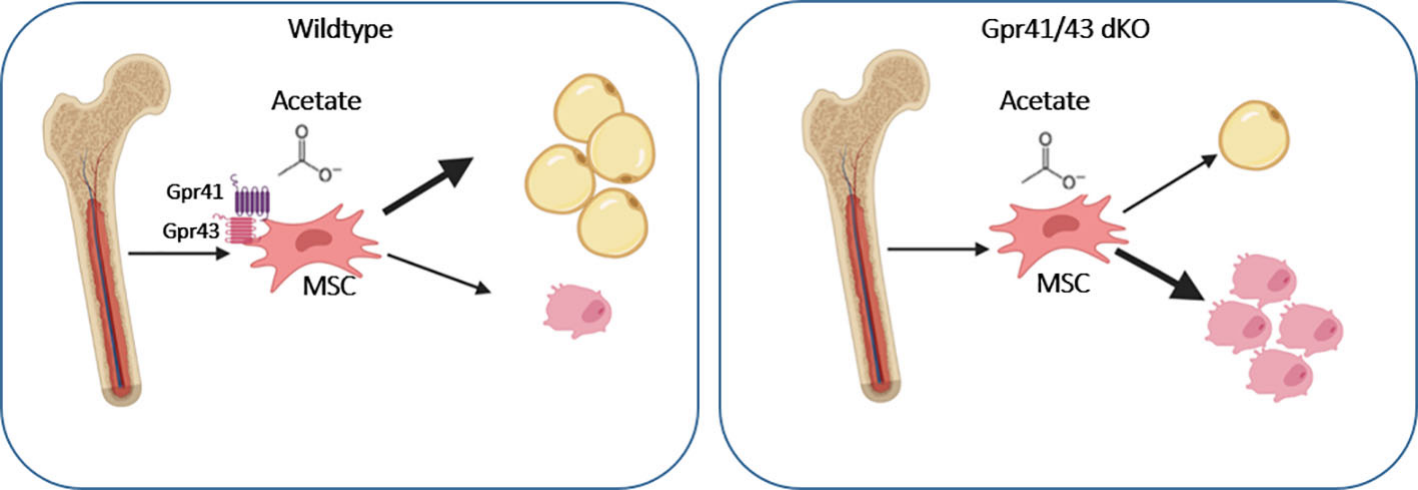
Graphical abstract showing the effect of acetate on WT and Gpr41/43 deficient mesenchymal stem cells (MSC). In the absent of short chain fatty receptors Gpr41 and Gpr43 treatment of MSC with acetate gives rise toward a greater extent osteoblastic differentiation compared to adipocytic differentiation.

In conclusion, our studies indicate that Gpr41/43-dependent signaling has a negative impact on bone formation in mice. Mechanistically, our data promote the concept that the SCFA acetate at physiological concentrations inhibits osteoblasts but promotes adipogenic differentiation of mesenchymal stem cells through Gpr41/43-dependent signaling (**Figure 7**). This signaling pathway could represent a new target for osteoanabolic therapy strategies to promote bone health.

## Data Availability

The data that support the findings of this study are available in GenBank with the accession number PRJNA1158734. These data can be accessed at https://www.ncbi.nlm.nih.gov/sra/PRJNA1158734.
