# Corrigendum: The short-chain fatty acid receptors Gpr41/43 regulate bone mass by promoting adipogenic differentiation of mesenchymal stem cells

**DOI:** 10.3389/fendo.2024.1528968

**Published:** 2025-01-07

**Authors:** Friederike Behler-Janbeck, Anke Baranowsky, Timur A. Yorgan, Michelle Y. Jaeckstein, Anna Worthmann, Marceline M. Fuh, Karthikeyan Gunasekaran, Gisa Tiegs, Michael Amling, Thorsten Schinke, Joerg Heeren

**Affiliations:** ^1^ Department of Biochemistry and Molecular Cell Biology, University Medical Center Hamburg-Eppendorf, Hamburg, Germany; ^2^ Department of Trauma and Orthopaedic Surgery, University Medical Center Hamburg-Eppendorf, Hamburg, Germany; ^3^ Department of Osteology and Biomechanics, University Medical Center Hamburg-Eppendorf, Hamburg, Germany; ^4^ Institute of Experimental Immunology and Hepatology, University Medical Center Hamburg-Eppendorf, Hamburg, Germany

**Keywords:** G protein-coupled receptors, Gpr41/43, short-chain fatty acids (SCFAs), acetate, osteoblasts, bone formation, adipogenesis

In the published article, there was an error in **Figure 5** as published. The wrong image in **Figure 5G** was unintentionally inserted due to a copy and paste error when compiling the revised version of the manuscript. The corrected **Figure 5** and its caption appear below.

**Figure 5:**
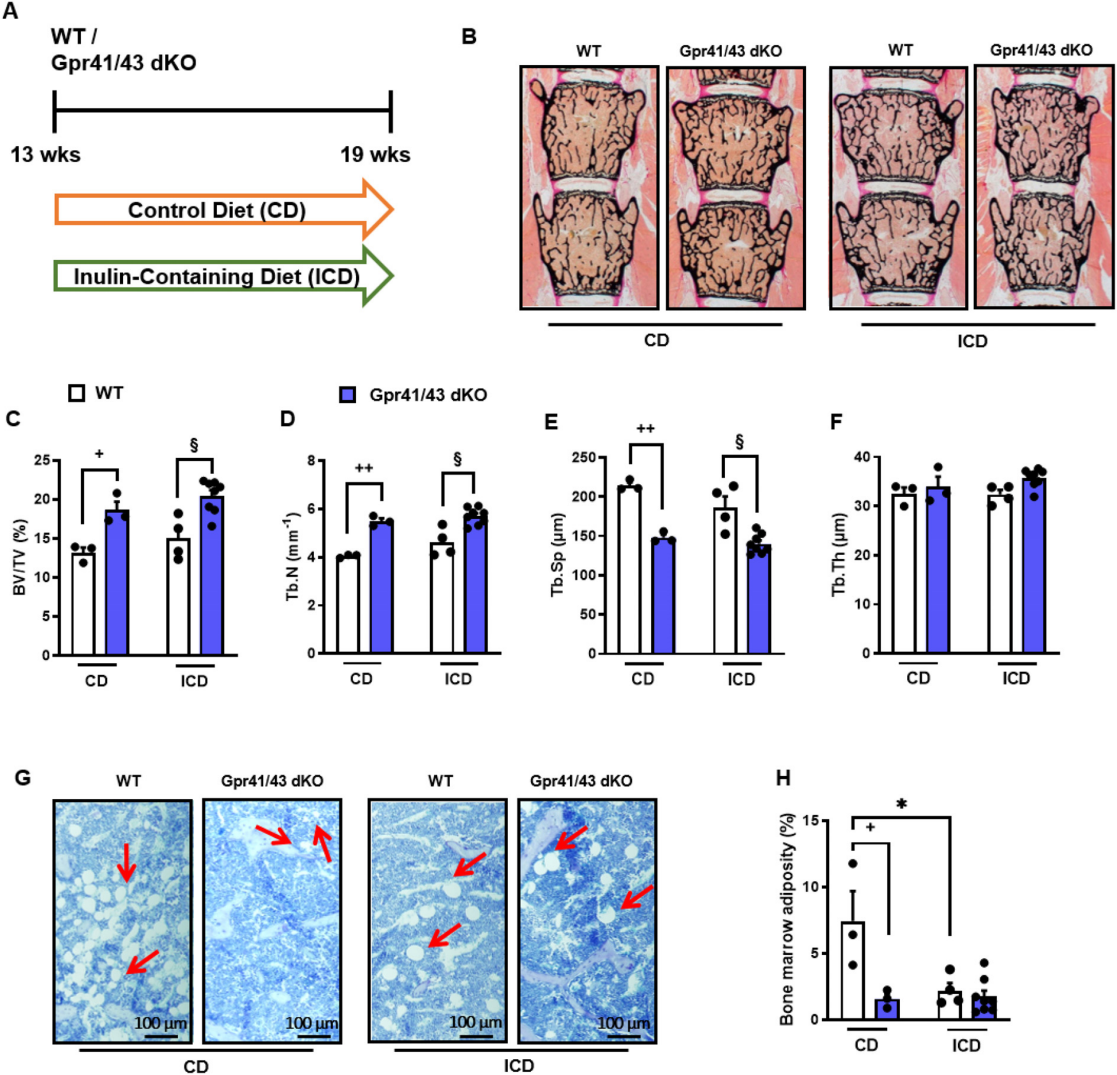
Inulin-containing diet decreases bone marrow adiposity. **(A)** Study design: 13 weeks old male WT (white bars) and Gpr41/43 dKO mice (blue bars) were fed an inulin-containing diet (ICD) or a respective control diet (CD) for 6 weeks. **(B)** Representative undecalcified histological sections and of vertebral bodies from 19 weeks old male WT and Gpr41/43 dKO mice fed an inulin-containing diet (ICD) or a respective control diet (CD). **(C–F)** Histomorphometric evaluation of trabecular bone parameters in the same sections. **(C)** Bone volume per tissue volume, **(D)** trabecular numbers (Tb.N), **(E)** trabecular spacing (Tb.Sp), **(F)** trabecular thickness (Tb.Th). **(G)** Representative images of toluidine blue stained tibia sections and red arrows indicate bone marrow adipocytes. **(H)** Quantification of bone marrow adiposity from the same section. Data were shown as dot plots with median values indicated as horizontal bars ± SEM ^+^p < 0.05 WT CD vs. Gpr41/43 dKO CD, ^++^p < 0.01 WT CD vs. Gpr41/43 dKO CD, *p < 0.05 WT CD vs. WT ICD, ^#^p < 0.05 Gpr41/43 dKO CD vs. Gpr41/43 dKO ICD, ^§^p < 0.05 WT ICD vs. Gpr41/43 dKO ICD, determined by two-way ANOVA. WT, CD: n=3, Gpr41/43, CD: n=3, WT, ICD: n=4, Gpr41/43 dKO, ICD: n=8.

The authors apologize for this error and state that this does not change the scientific conclusions of the article in any way. The original article has been updated.

