# Supplementary material for: The short-chain fatty acid receptors Gpr41/43 regulate bone mass by promoting adipogenic differentiation of mesenchymal stem cells
Source: Front Endocrinol (Lausanne). 2024 Sep 19;15:1392418. doi: 10.3389/fendo.2024.1392418 (PMC11446854; doi:10.3389/fendo.2024.1392418)
Supplement: Supplementary file 1 [file DataSheet1.pdf]

Supplementary Material

The short-chain fatty acid receptors Gpr41/43 regulate bone mass by promoting adipogenic differentiation of mesenchymal stem cells

Friederike Behler-Janbeck[1*], Anke Baranowsky[2], Timur A. Yorgan[3], Michelle Y. Jaeckstein[1], Anna Worthmann[1], Marceline M. Fuh[1], Karthikeyan Gunasekaran[1], Gisa Tiegs[4], Michael Amling[3], Thorsten Schinke[3], Joerg Heeren[1**]**

[1]Department of Biochemistry and Molecular Cell Biology, University Medical Center Hamburg-Eppendorf, Hamburg, Germany

[2]Department of Trauma and Orthopaedic Surgery, University Medical Center Hamburg-Eppendorf, Hamburg, Germany

[3]Department of Osteology and Biomechanics, University Medical Center Hamburg-Eppendorf, Hamburg, Germany

[4]Institute of Experimental Immunology and Hepatology, University Medical Center Hamburg-Eppendorf, Hamburg 20246, Germany

* **Correspondence:** Friederike Behler-Janbeck: f.behler-janbeck@uke.de

****contributed equally**

1 Supplementary Figures and Tables

1.1 Supplementary Figures

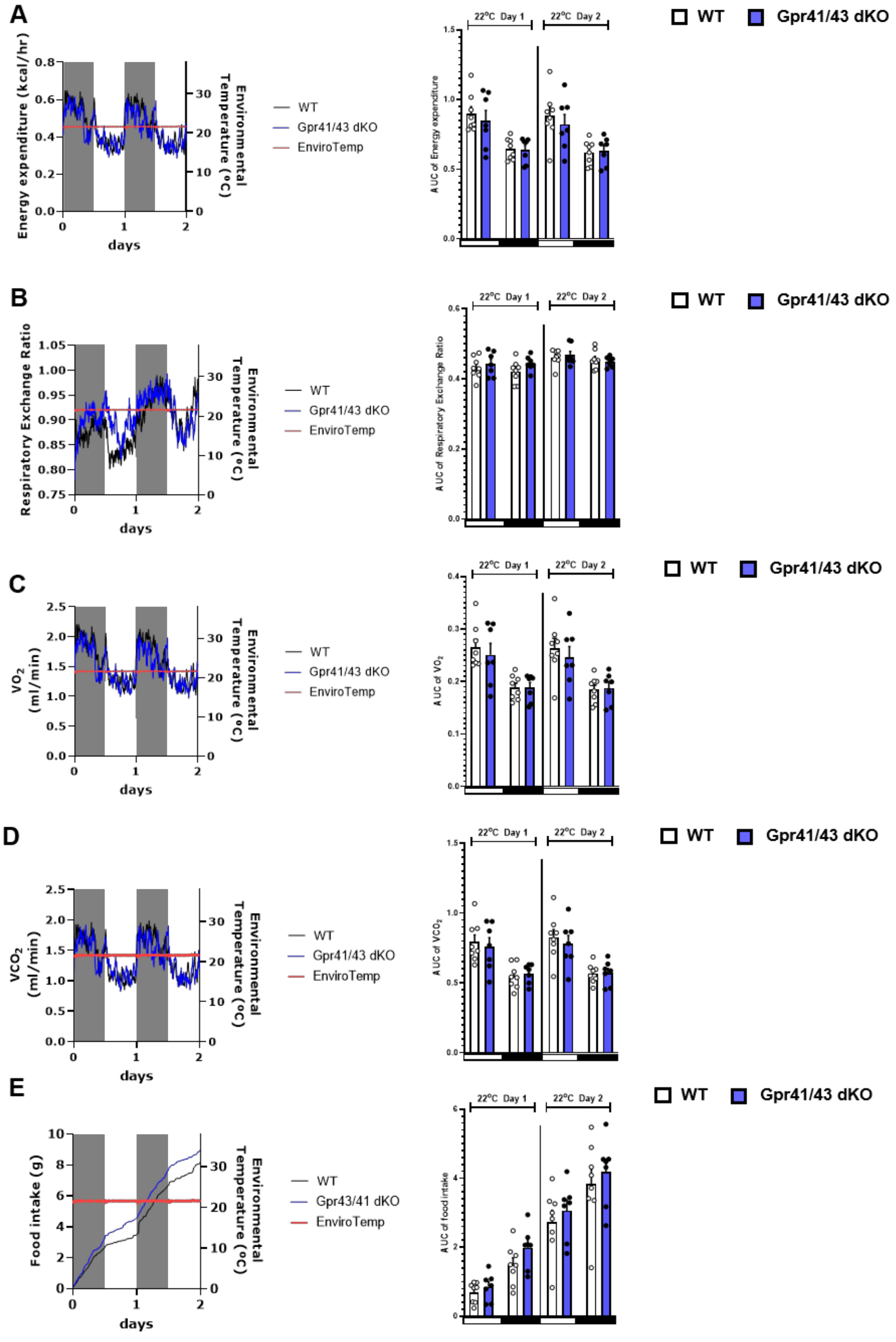

Supplementary Figure 1. No difference in energy expenditure parameters and food intake between GPR43/41 KO (blue bars) and their wild type littermates (white bars). (A) Energy expenditure (kcal/hr), (B) Respiratory exchange ratio, (C) VO_2 (ml/min), (D) VCO_2 (ml/min) and (E) food intake (grams) with their respective area under the curve (AUC).

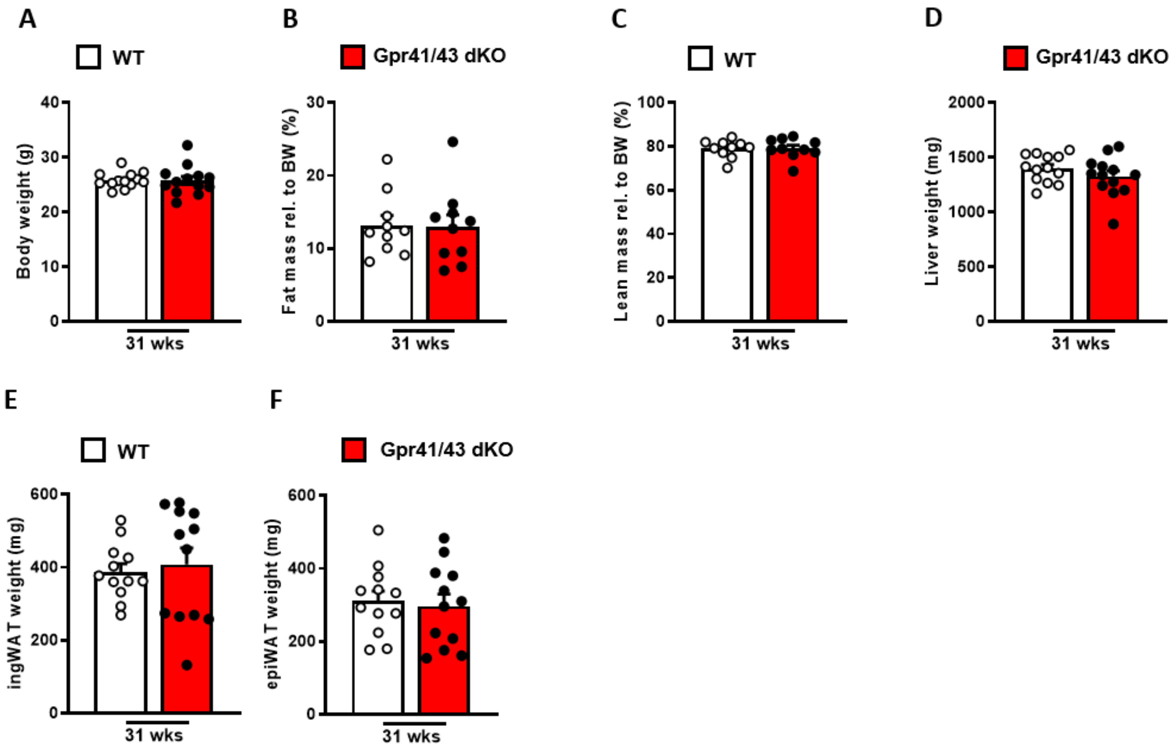

Supplementary Figure 2 Investigation of metabolic phenotype in Gpr41/43 double-deficient female mice. WT (white bars) and Gpr41/43 dKO (red bars) mice were used to investigate body composition and organ weights at 31 weeks of age (n=13 WT, n=9-13 Gpr41/43 dKO). (A) body weight, (B) fat mass rel. to body weight (BW), (C) lean mass rel. to BW, (D) liver weight, (E) inguinal white adipose tissue (ingWAT), (F) epididymal white adipose tissue (epiWAT). Data were shown as dot plots with median values indicated as horizontal bars ± SEM analyzed by Student's t-test.

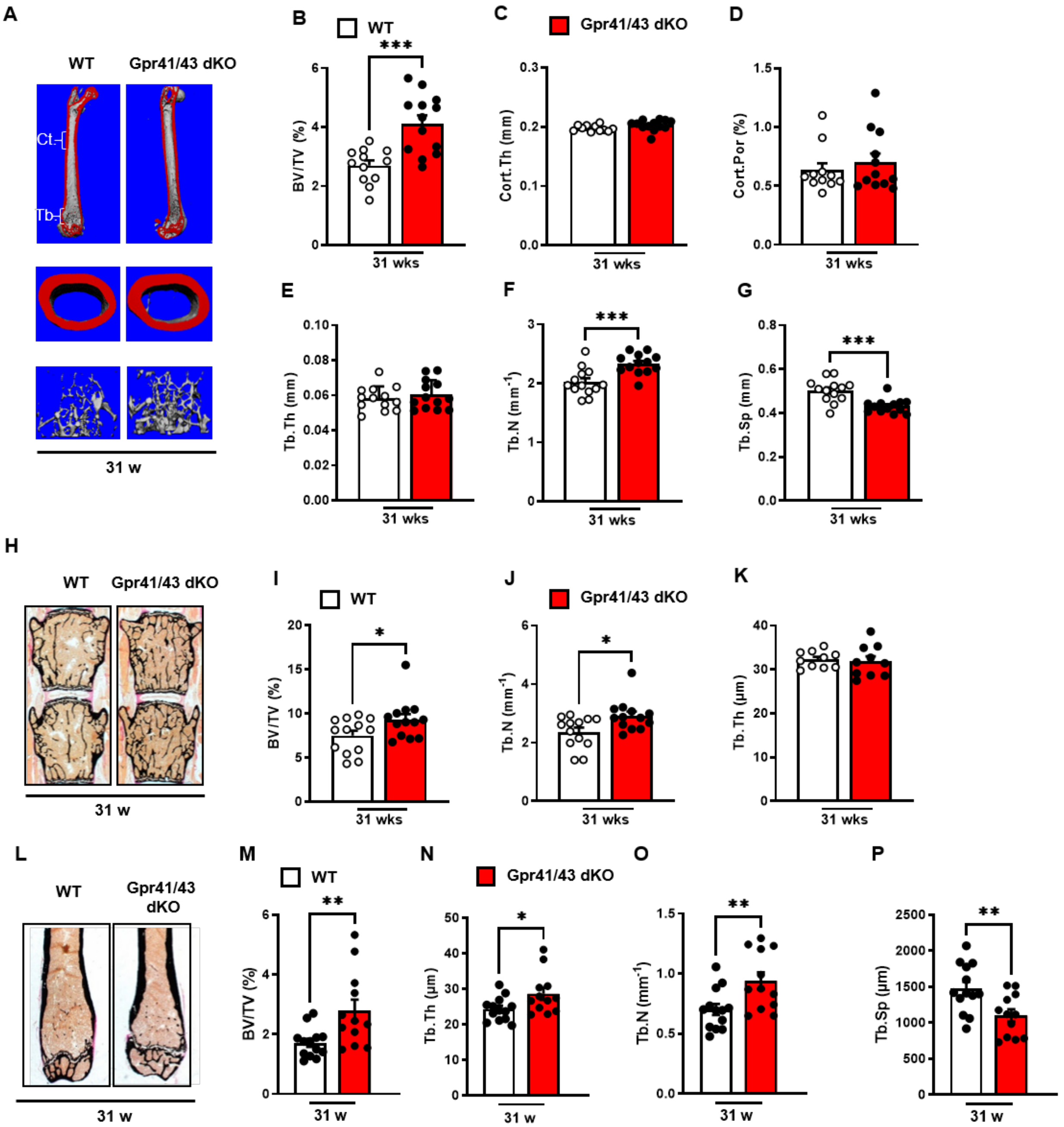

Supplementary Figure 3. Skeletal phenotype of Gpr41/43 double-deficient female mice. (A) Representative µCT images of femora from WT (white bars) and Gpr41/43 dKO mice (red bars). µCT-based quantification of 31 weeks old male WT and Gpr41/43 dKO mice (n=10-13 WT, n=10-13 Gpr41/43 dKO) showing whole femurs (top, the virtual cut plane appears red), cortical (Ct., middle) and trabecular bone (Tb., bottom). (B) trabecular bone volume per tissue volume (BV/TV), (C) cortical thickness (Cort.Th), (D) cortical porosity (Cort.Por), (E) trabecular thickness (Tb.Th), (F) trabecular numbers (Tb.N), (G) trabecular spacing (Tb.Sp). (H) Representative images of undecalcified histological sections of Von Kossa/van Gieson stained vertebral bodies from and 31 weeks old female WT and Gpr41/43 dKO mice. (I-K) Histomorphometric evaluation of trabecular bone parameters in

the same sections. (I) Bone volume per tissue volume, (J) trabecular numbers (TB.N) and (K) trabecular thickness (Tb.Th). (L) Representative images of undecalcified histological sections of Von Kossa/van Gieson stained femora from 31 weeks old female WT and Gpr41/43 dKO mice. (M-P) Histomorphometric evaluation from undecalcified histological sections of Von Kossa/van Gieson of femur sections. (M) Bone volume per tissue volume, (N) trabecular thickness (Tb.Th), (O) trabecular numbers (TB.N), (P) trabecular spacing (Tb.Sp). Data were shown as dot plots with median values indicated as horizontal bars ± SEM analyzed by Student's t-test. *p < 0.05 vs. Gpr41/43 dKO, **p < 0.01 vs. Gpr41/43 dKO.

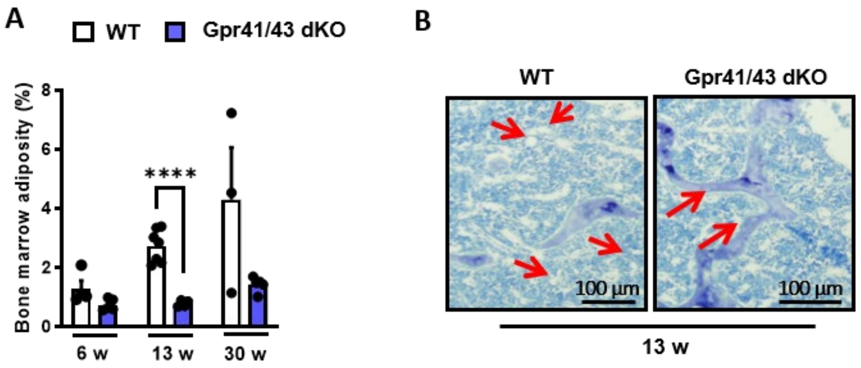

Supplementary Figure 4. Quantification of bone marrow adiposity in WT and Gpr41/43 dKO mice. (A) Quantification of bone marrow adiposity bone marrow adiposity in tibia of WT (white bars) and Gpr41/43 dKO (blue bars). (B) Representative images of toluidine blue stained proximal tibiae sections of 13 weeks old male WT and Gpr41/43 dKO mice. Red arrows indicate adipocytes. Data are shown as as dot plots with median values indicated as horizontal bars ± SEM. ***p < 0.001 determined by Student's t-test. WT: n=3-7, Gpr41/43 dKO: n=4-5.

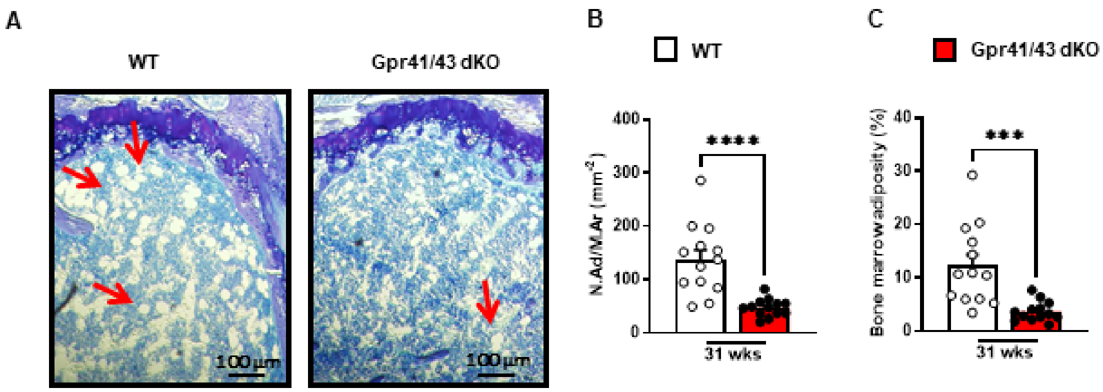

Supplementary Figure 5. Quantification of bone marrow adiposity in WT and Gpr41/43 dKO mice. Representative images of toluidine blue stained proximal tibiae sections of 31 weeks old female WT (white bars) and Gpr41/43 dKO mice (red bars). Red arrows indicate bone marrow adipocytes. (B) Quantification of numbers of bone marrow adipocytes per bone marrow area (N.Ad/M.Ar) and (C) bone marrow adiposity. Data are shown as as dot plots with median values indicated as horizontal bars ± SEM. $**p < 0.01$,$***p < 0.001$, $****p < 0.0001$ determined by Student's t-test. WT: n=13, Gpr41/43 dKO: n=13.

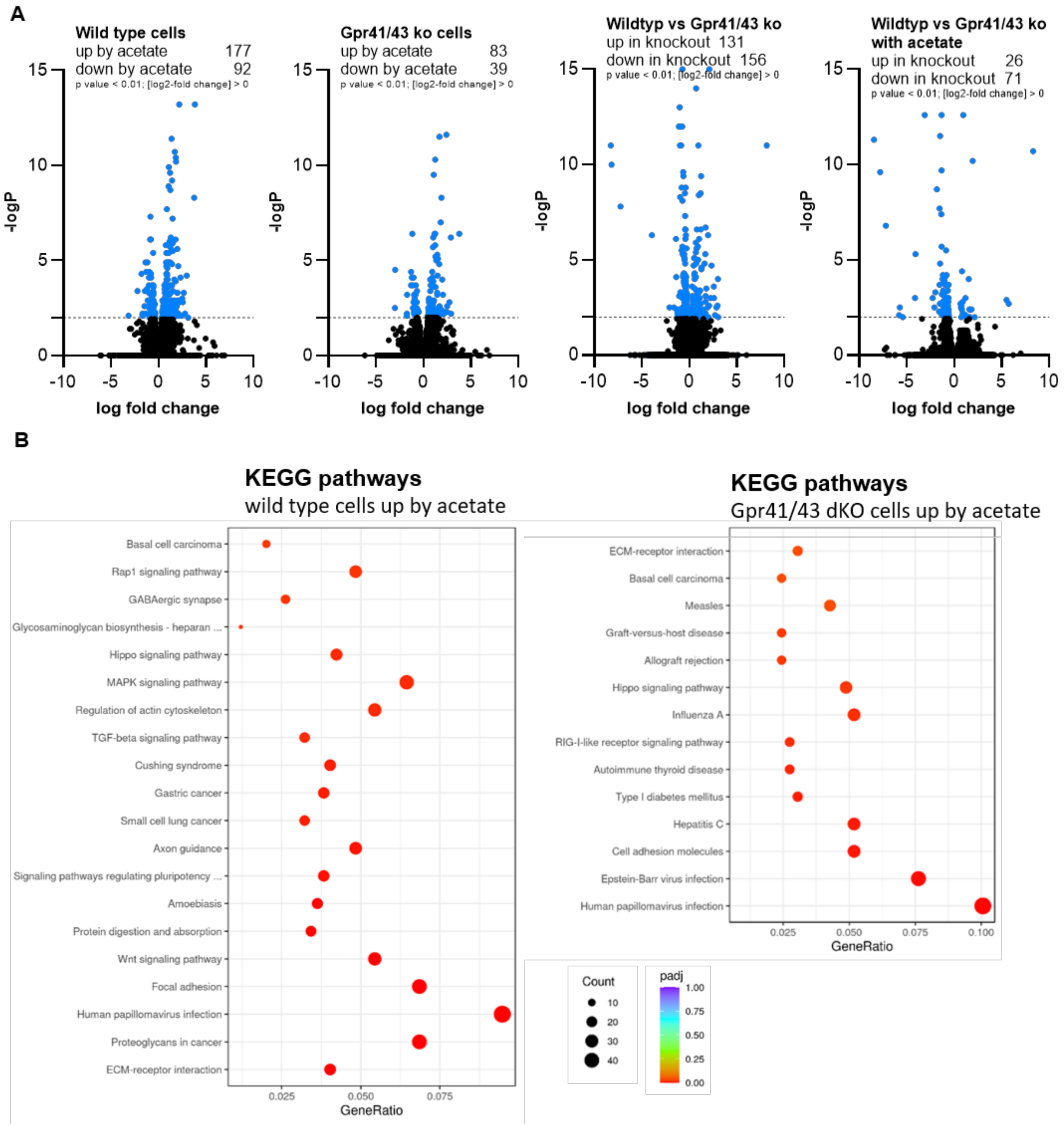

Supplementary Figure 6. Genome-wide expression analysis: RNA sequencing of wild type and Gpr41/43-deficient bone marrow cells induced to differentiate into osteoblasts in the absence or presence of acetate. (A) Volcano plot presentation of genes with an adjusted p-value < 0.01 and |log2(FoldChange)| > 0 were considered as differentially expressed. (B) KEGG pathways of WT and Gpr41/43 dKO cells upregulated by acetate.

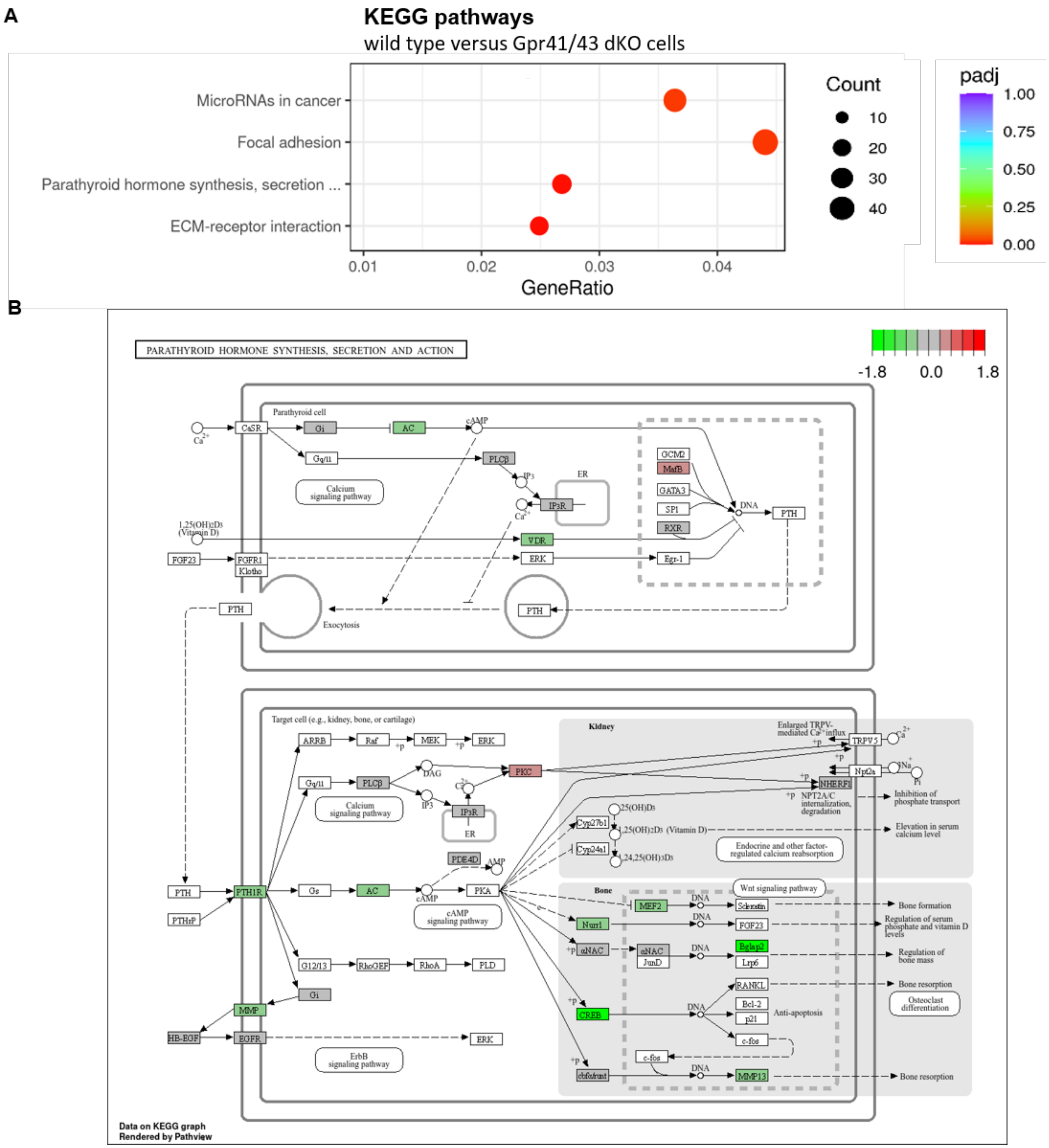

Supplementary Figure 7. (A) KEGG pathways of WT and Gpr41/43 dKO cells. (B) Direct comparison of WT and Gpr41/43 dKO osteoblasts suggests that parathyroid hormone receptor signaling is modulated.

Supplementary table 1

Supplementary Table 1. The gene expression pattern of downregulated and induced adipocyte and osteoblast marker genes, as determined by Gene Chip hybridization comparing control and acetate-treated WT osteoblasts at day 5 of osteoblastic differentiation.

control (log2)	acetate (log2)	fold change	gene
14.8	4.94	-930.95	*Cdsn*
13.56	4.37	-586.59	*Car12*
14.26	6.50	-217.58	*Ptprz1*
12.63	5.33	-157.21	*Ndufa4l2*
11.82	4.53	-155.95	*Slitrk6*
13.28	6.75	-92.66	*Aldh1l2*
13.7	7.21	-90.1	*Phex*
14.09	7.91	-72.54	*Ptgs2*
11.33	5.15	-72.37	*Wif1*
13.25	7.16	-68.34	*Cgref1*
9.89	4.01	-58.85	*Entpd3*
12.38	6.54	-57.1	*Pcsk6*
10.98	5.55	-43.06	*Egln3*
16.67	11.41	-38.43	*Slc13a5*
12.46	7.24	-37.43	*Ddit3*
12.59	7.39	-36.73	*Adgrd1*
11.5	6.42	-33.84	*Loxl4*
12.8	7.87	-30.37	*Ddit3*
10.52	5.84	-25.62	*Kcnk1*
8.95	4.28	-25.55	*Trib3*
14.68	10.08	-24.27	*Ero1l*
14.33	9.75	-23.93	*Tnn*
14.39	9.90	-22.47	*Gm16439*
13.57	9.14	-21.68	*Snord123*
11.59	7.20	-20.97	*Slc6a9*
3.28	7.51	18.78	*Fpr1*
5.27	9.57	19.63	*Tmem178*
6.25	10.59	20.29	*Ccl12*
9.36	13.74	20.79	*Mki67*
5.97	10.36	21.03	*Susd2*
8.57	13.00	21.57	*Errfi1*
9.31	13.75	21.7	*Pf4*
5.18	9.88	26.05	*Gsta4*
6.66	11.39	26.46	*Esm1*
7.04	11.77	26.51	*Epas1*
6.3	11.04	26.78	*F2r*
4.43	9.22	27.61	*Efemp1*
8.07	13.03	31.06	*Ccl6*
6.69	11.72	32.61	*Eno3*
6.47	11.53	33.26	*Ppp1r14a*
9.71	14.80	34.16	*Sfrp1*
9.81	14.96	35.58	*Cxcl12*
6.68	11.83	35.63	*Arrdc4*
9.04	14.33	39.26	*Ch25h*
8.35	13.92	47.59	*Has2*
6.44	12.08	49.93	*Ms4a4d*
6.89	12.79	59.35	*Pi15*
3.09	9.03	61.42	*Pard3bos2*
7.78	14.03	75.98	*Stc1*
8.42	15.52	137.3	*Aldh1a2*

Supplementary Table 2. KEGG pathway analysis and differentially expressed genes, determined by bulk RNA sequencing comparing control and acetate-treated WT osteoblasts as well as Gpr41/43 double knockout osteoblasts at day 5 of osteoblastic differentiation (see Excel file).
